# Supplementary material for: Subsurface oxygen defects electronically interacting with active sites on In2O3 for enhanced photothermocatalytic CO2 reduction
Source: Nat Commun. 2022 Jun 9;13:3199. doi: 10.1038/s41467-022-30958-5 (PMC9184511; doi:10.1038/s41467-022-30958-5)
Supplement: Supplementary file 1 — Supplementary Information [file 41467_2022_30958_MOESM1_ESM.pdf]

Subsurface Oxygen Defects Electronically Interacting with Active Sites on In_2O_3 for Enhanced Photothermocatalytic CO_2 Reduction

Weiqin Wei[1], Zhen Wei[2], Ruizhe Li[1], Zhenhua Li[3], Run Shi[3], Shuxin Ouyang[1*], Yuhang Qi[4], David Lee Philips[2], Hong Yuan[1]

[1] Key Laboratory of Pesticide and Chemical Biology of Ministry of Education, College of Chemistry, Central China Normal University, Wuhan 430079, China

[2] Department of Chemistry, University of Hong Kong, Pokfulam Road, Hong Kong SAR, P. R. China

[3] Key Laboratory of Photochemical Conversion and Optoelectronic Materials, Technical Institute of Physics and Chemistry, Chinese Academy of Sciences, Beijing, 100190, P. R. China

[4] Chemical Engineering Institute, Hebei University of Technology, Tianjin 300131, China

*Author to whom correspondence should be addressed.
Email: oysx@mail.ccnu.edu.cn

Contents

Supplementary Methods 4

Supplementary Figures 7

Supplementary Fig. 1 XRD pattern and TG curve of precursor $In(OH)_3$ 7

Supplementary Fig. 2 H_2-TPR patterns of In_2O_3 and In-Em In_2O_3 8

Supplementary Fig. 3 XRD patterns of In_2O_3 and In-Em In_2O_3. 9

Supplementary Fig. 4 XPS survey spectra of In_2O_3 and In-Em In_2O_3 10

Supplementary Fig. 6 N_2 adsorption-desorption isothermal curves and pore size distribution curves of In_2O_3 and In-Em In_2O_3. 12

Supplementary Fig. 7 Magnified XRD patterns. 13

Supplementary Fig. 8 HRTEM images of In_2O_3 14

Supplementary Fig. 9 AFM image of In-Em In_2O_3. 15

Supplementary Fig. 10 AFM image of some separated In nanoparticles 16

Supplementary Fig. 11 In_{3d} XPS spectra of In_2O_3 and In-Em In_2O_3 17

Supplementary Fig. 12 A histogram summarizing the oxygen species distribution in In_2O_3 and In-Em In_2O_3 18

Supplementary Fig. 13 CO_2-TPD profiles of In_2O_3 and In_2O_3 with HCl etching 19

Supplementary Fig. 14 Raman spectra of In_2O_3 and In-Em In_2O_3 20

Supplementary Fig. 15 Analysis of charge balance around oxygen defects 21

Supplementary Fig. 16 CO mass/area specific activities and CO_2 conversions over In_2O_3 and In-Em In_2O_3 22

Supplementary Fig. 17 Temperature profiles. 23

Supplementary Fig. 18 Maintenance of oxygen defects 24

Supplementary Fig. 19 Characterization of In-Em In_2O_3-spent(10) 25

Supplementary Fig. 20 XRD patterns of In_2O_3 and In_2O_3 spent 26

Supplementary Fig. 21 Performances over In-Em In_2O_3 and In-Em $In_2O_3(H_2O_2)$. 27

Supplementary Fig. 22 Characterization of of In-Em $In_2O_3(H_2O_2)$ 28

Supplementary Fig. 23 XRD patterns of metallic In, In-Em In_2O_3 and In-Sup In_2O_3 29

Supplementary Fig. 24 FT-IR spectra of In_2O_3 and In-Sup In_2O_3. 30

Supplementary Fig. 25 Light effect. 31

Supplementary Fig. 26 CO_2-TPD profile of In-Sup In_2O_3 32

Supplementary Fig. 27 Variable temperature ESR spectra of In_2O_3 and In-Em In_2O_3 33

Supplementary Fig. 28 Positron annihilation discrete spectra of In_2O_3 and In-Em In_2O_3 ... 34
Supplementary Fig. 29 Positron trapping rates and trapping rate constants over In_2O_3 and In-Em In_2O_3 ... 35
Supplementary Fig. 30 Fluorescence spectra ... 36
Supplementary Fig. 31 Fourier-transformed R space and K space EXAFS spectra of In ... 37
Supplementary Fig. 32 The corresponding R space EXAFS fitting curves of In ... 38
Supplementary Fig. 33 The corresponding K space EXAFS fitting curves of In ... 39
Supplementary Fig. 34 Wavelet transform analysis ... 40
Supplementary Fig. 35 LDOS of defective In_2O_3 and metallic In ... 41
Supplementary Fig. 36 LDOS of In_2O_3 and In-In_2O_3 ... 42
Supplementary Fig. 37 Charge distribution derived from wave function of energy levels at VBM of In_2O_3 and In-In_2O_3. ... 43
Supplementary Fig. 38 Performances over In-Em In_2O_3 with Fe^{3+} ... 44
Supplementary Fig. 39 The pictures of Nicolet iS50FTIR spectrometer ... 46
Supplementary Fig. 40 The adsorbate binding configurations on the surface of In_2O_3 ... 46
Supplementary Fig. 41 The adsorbate binding configurations on the surface of In-In_2O_3 .. 47
Supplementary Tables ... 48
Supplementary Table 1 Specific surface areas of In_2O_3 and In-Em In_2O_3 ... 48
Supplementary Table 2 Crystal size ... 49
Supplementary Table 3 Lifetimes and relative intensities of positrons in positron annihilation spectra of In_2O_3 and In-Em In_2O_3 ... 50
Supplementary Table 4 Performance comparison of different catalysts for CO_2 hydrogenation into CO ... 51
Supplementary Table 5 CO_2 amount adsorbed measured from CO_2-TPD patterns ... 53
Supplementary Table 6 EXAFS fitting parameters at the In K-edge for various samples ... 54
Supplementary References ... 55

Supplementary Methods

Proof method for CO production calculation in the sealed vessel

First, a mixture gas of $CO/H_2/N_2$ (the mole ratio= 1/3/1) was injected into a sealed vessel to reach a total pressure of 0.18 MPa. Then, the corresponding peak area ($A_{standard}$) of CO was obtained via gas chromatography.

For the standard sample, the parameters meet the equation:

$$n_{standard}/n_{standard'} = 20\%P_{total}V_{vessel}/20\%P_0V_{quantitative\ loop} = P_{total}V_{vessel}/P_0V_{quantitative\ loop} \quad (1)$$

where $n_{standard}$ is the CO mole quantity in the sealed vessel, $n_{standard'}$ is the CO mole quantity in the quantitative loop, P_{total} is the total pressure in the sealed vessel, V_{vessel} is the volume of the sealed vessel, P_0 is the normal pressure and $V_{quantitative\ loop}$ is the volume of the quantitative loop.

After photothermal catalysis, the corresponding peak area (A) of CO was also obtained via gas chromatography. It is noted that the total pressure is always the same since the stoichiometric numbers before and after reaction are constant.

For the reaction sample, the parameters meet the equation:

$$n/n' = x\%P_{total}V_{vessel}/x\%P_0V_{quantitative\ loop} = P_{total}V_{vessel}/P_0V_{quantitative\ loop} \quad (2)$$

where n is CO mole quantity in the sealed vessel, n' is the CO mole quantity in the quantitative loop, $x\%$ is the percentage of CO generated, P_{total} is the total pressure in the sealed vessel, V_{vessel} is the volume of the sealed vessel, P_0 is the normal pressure and $V_{quantitative\ loop}$ is the volume of the quantitative loop.

So, the equation is as following:

$$n_{standard}/n_{standard'} = n/n' \quad (3)$$

Meanwhile, the equation is as following:

$$n_{standard'}/n' = A_{standard}/A \quad (4)$$

Finally we can get the equation:

$$n_{standard}/n = A_{standard}/A \quad (5)$$

In-situ FT-IR spectra

In-situ FT-IR spectra were carried out using a Nicolet iS50FTIR spectrometer (Thermo, U.S.A.) with a liquid-nitrogen-cooled MCT-A detector. The experimental setup is displayed in Supplementary Fig. 39.

Put about 100 mg of the sample into the sample tank of the stainless-steel vacuum chamber with two pieces of copper mesh at the bottom and compact it. Set up the device according to the setup in the picture above, turn on the condensate water, and add liquid nitrogen toward the liquid N_2 added inlet. After degassing, the sample was heated to 150°C and kept for 2 hours to remove the gas adsorbed on the sample surface. After that, the temperature dropped to room temperature. Before the experiment, the DRIFTS accessory optics were aligned and optimized. Inject a certain amount of CO_2 or CO_2/H_2 gas into the reaction cell. Data collection consisted of 64 scans per spectrum with a resolution of 4 cm^{-1}.

For the CO_2 adsorption measurement, the adsorption of the intermediates over In-Em In_2O_3 failed to be measured due to the negligible amount of their buildup concentration during the experiments. The bands at 3500~4000 cm^{-1} correspond to a combination mode ($\nu_1 + \nu_3$) of adsorbed CO_2, so the change in the adsorption amount was used to evaluate the capability of the CO_2 adsorption[1].

Curve of rate constant (ln *k*) vs. 1/*T*

According to the Gibbs-Helmholtz equation:

$$[d(G/T)/dT]_p=-H/T^2 \quad (6)$$

where G is Gibbs free energy, H is Helmholtz energy and T is temperature, for each reactant in the system corresponding to a chemical reaction $0=\Sigma v_B B$, the derived equation:

$$d(\Delta_r G_m/T)/dT=-\Delta_r H_m/T^2 \quad (7)$$

where $\Delta_r G_m$ is Gibbs free energy variation of the reaction under specified conditions, $\Delta_r H_m$ is Helmholtz energy variation of the reaction under specified conditions and T is temperature, or

$$d\ln K/dT=\Delta_r H_m/T^2 \text{ (Van't Hoff equation)} \quad (8)$$

where K is equilibrium constant, $\Delta_r H_m$ is Helmholtz energy variation of the reaction under specified conditions and T is temperature, is acquired in the standard state, which suggests that there is temperature effect on the equilibrium constant. Then, we can get the equation:

$$\ln K=4950.1/T+28.17 \quad (9)$$

where it was assumed that $\Delta_r H_m$ remains fairly constant because specific heat capacity variation ($\Delta_r C_{p,m}$) is small enough. Based on the CO_2 conversion rate (a) and the feed gases CO_2/H_2 concentration percentages (1/3), the real reaction in one hour only proceeds to

$$J=a^2/(1.5-a)(4.5-a) \quad (10)$$

where J is reaction proceeding degree during the process and a is CO_2 conversion rate, before the equilibrium state. Therefore, it can be thought that it is far from the equilibrium state, and CO_2 conversion rate for one hour approximately presents a linear correlation with the reaction rate constant because the surface catalytic process is in a zero-grade mode (linear relationship between C_{CO} and t), namely

$$a=k/C_0 \text{ (based on } dC_{CO}/dt=k) \quad (11)$$

where a is CO_2 conversion rate, k is rate constant, C_0 is initial concentration of CO_2, C_{CO} is concentration of CO and t is reaction time. Besides, according to the Arrhenius equation:

$$d\ln k/dT=E_a/RT^2 \quad (12)$$

where k is rate constant, T is temperature, E_a is activation energy and R is molar gas constant, a relatively unchanged activation energy E_a with the temperatures involved in the experiment, the apparent activation energies E_a at different temperatures can be compared in the curve. It is noteworthy that the apparent activation energy in multiple reactions has no explicit physical meaning, only to present the reaction pathway difference with the reaction proceeding. If the CO_2 reduction over the catalysts undergoes invariant reaction pathways, the curve of ln k vs. $1/T$ would present a linear correlation.

Percentage calculations of surface oxygen defects

Through the unit cell parameters, we take 0.1012*0.1012*0.1012 nm^3 ($1.036*10^{-3}$ nm^3) as the calculated volume. The volume unit contains 50 oxygen atoms, which means that 1 nm^3 volume contains $4.826*10^4$ oxygen atoms. According to the CO_2 chemisorption amount and the BET specific surface area, the number of oxygen defects per unit area can be obtained:

In_2O_3: 0.375 mmol g^{-1}/101.25 m^2 g^{-1}=0.00370 mmol m^{-2}=2.227 nm^{-2};

In-Em In_2O_3: 0.0012 mmol g^{-1}/6.94 m^2 g^{-1}=0.000173 mmol m^{-2}=0.104 nm^{-2}.

The percentages of the oxygen defects on the surface and the subsurface in the XPS spectra are: 28.8% (In_2O_3), 26,0% (In-Em In_2O_3), that is, 6949.44 nm^{-3} and 6273.8 nm^{-3}.

Therefore, the surface oxygen defects/total oxygen defects=0.032% (In_2O_3) and 0.0017% (In-Em In_2O_3).

Calculations of the positron trapping rate (κ) and the positron trapping rate constant (k)

The calculation is based on that described previously in the literature[2].

$$\tau_B = (I_1/\tau_1 + I_2/\tau_2)^{-1} \quad (13)$$

$$\kappa = I_2/I_1(1/\tau_B + 1/\tau_2) \quad (14)$$

where τ_B is bulk lifetime, I_1 and I_2 are relative contents of the first and second component, respectively, τ_1 and τ_2 are the lifetimes of the first and second component, respectively.

According to

$$\kappa = k*I \quad (15)$$

where k is positron trapping rate constant and I is relative content, we can obtain the positron trapping rate constant. The total numbers of the annihilated positrons over In_2O_3 and In-Em In_2O_3 are the same (Supplementary Fig. 28), thus the relative intensities can be used as the concentration of the trapped positrons for comparison.

Periodic DFT calculations

All periodic DFT calculations were carried out with the Vienna ab initio simulation package (VASP)[3-4] using the Perdew-Burke-Ernzerhof (PBE) exchange-correlation functional[5] and the projector-augmented wave (PAW) potentials[6]. An energy cut-off of 400 eV and the Gaussian smearing width of 0.05 eV were employed for optimizing the In_2O_3 bulk and the (222) surface. The electronic energy of the supercell was converged to 10^{-6} eV, and the force on all of the unconstrained atoms was converged to 0.03 eV $Å^{-1}$. The Methfessel-Paxton smearing with the width of 0.2 eV was employed for the calculations of the metallic In (101) surface. The In_2O_3 (222) surface was built from the optimized In_2O_3 conventional unit cell depending on the results from previous works[7-9], which contain the lattice parameters of A = B = C = 10.22 Å. The stoichiometric and symmetric In_2O_3 (222) slab was constructed with the vacuum layer of 10 Å along the z direction to avoid interactions of the neighboring slab. The In_2O_3 (222) surface was modeled with a p(1 × 1) slab consisting of 48 O atoms and 32 In atoms, and was optimized using a Monkhorst-Pack[10] (2 × 2 × 1) k-point mesh. The supercell has a dimension of 14.45 Å × 14.45 Å × 15.04 Å. For the In (101) surface, the slab thickness was set to be 4 layers, and each contains 8 In atoms. Upon optimizing the oxygen and hydrogen adsorption on the In (101) surface, the bottom half layers were kept constrained at their bulk positions. And a Monkhorst-Pack[10] (2 × 4 × 1) k-point mesh was applied for the supercell containing the dimension of 12.10 Å × 6.53 Å × 23.25 Å. Barder charge analysis was used to further investigate the electron distributions on the In_2O_3(222) surface with an oxygen vacancy and the In(101) surface. Charge density distributions were shown with the isosurface value of 0.1 e$Å^{-3}$.

Supplementary Figures

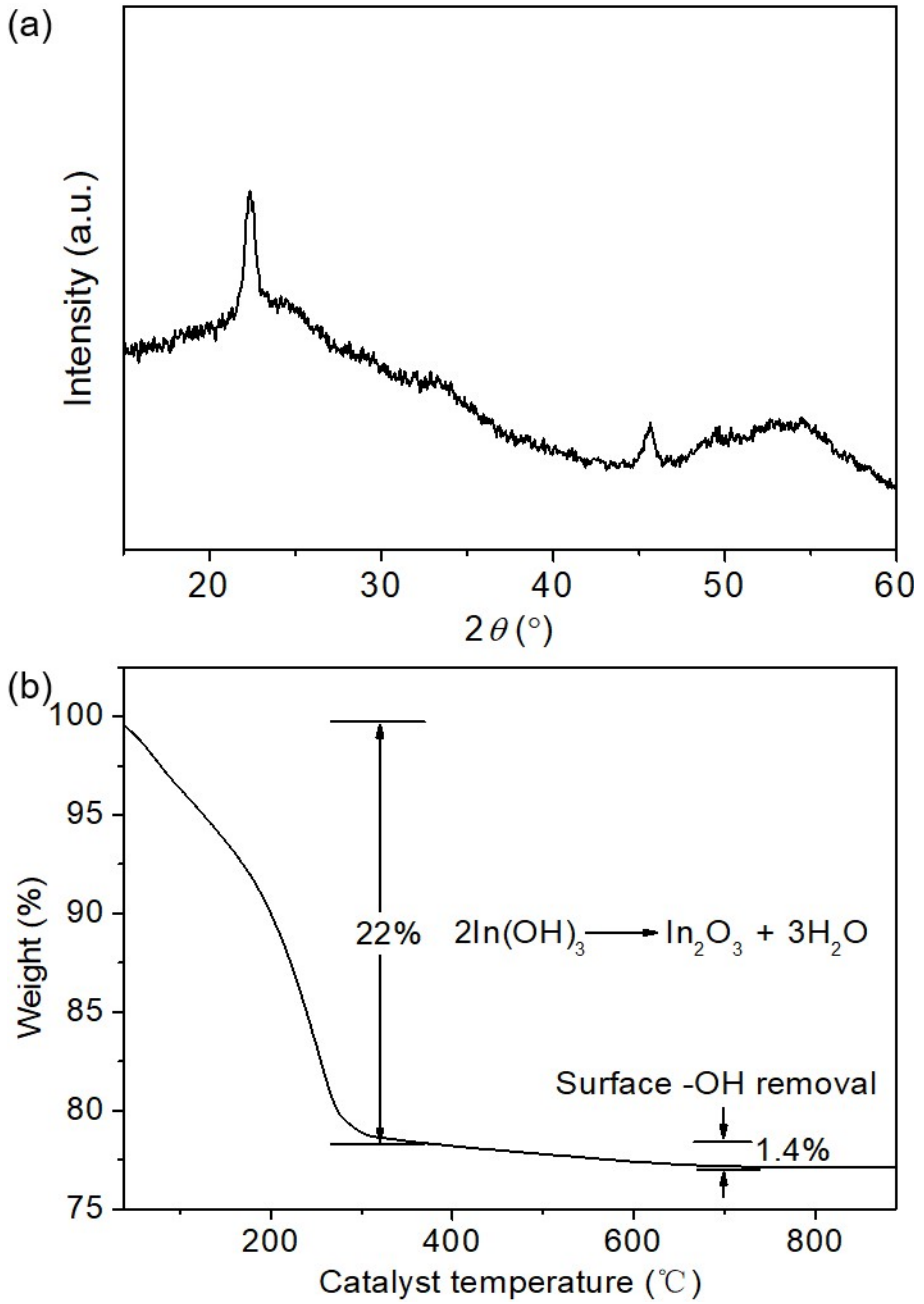

Supplementary Fig. 1 a XRD pattern of precursor In(OH)$_3$. The poor crystallinity of In(OH)$_3$ is demonstrated by the unobvious diffraction peaks due to the amorphous nature[11]. Some broad peaks at 22.2, 35.5, 45.5 and 56.3° approach to the JADE data of In(OH)$_3$ (JCPDS Card 16-0161). **b** TG curve of In(OH)$_3$ in air flow at 15 ml min^{-1}. At the temperature range from 35 to 300°C, In(OH)$_3$ starts losing its weight and the quality is reduced by 22% since In(OH)$_3$ is transformed into In$_2$O$_3$. This matches well with the theoretical mass loss of 16% expected in the formation of In$_2$O$_3$ from In(OH)$_3$: 2In(OH)$_3$ → In$_2$O$_3$ + 3H$_2$O.

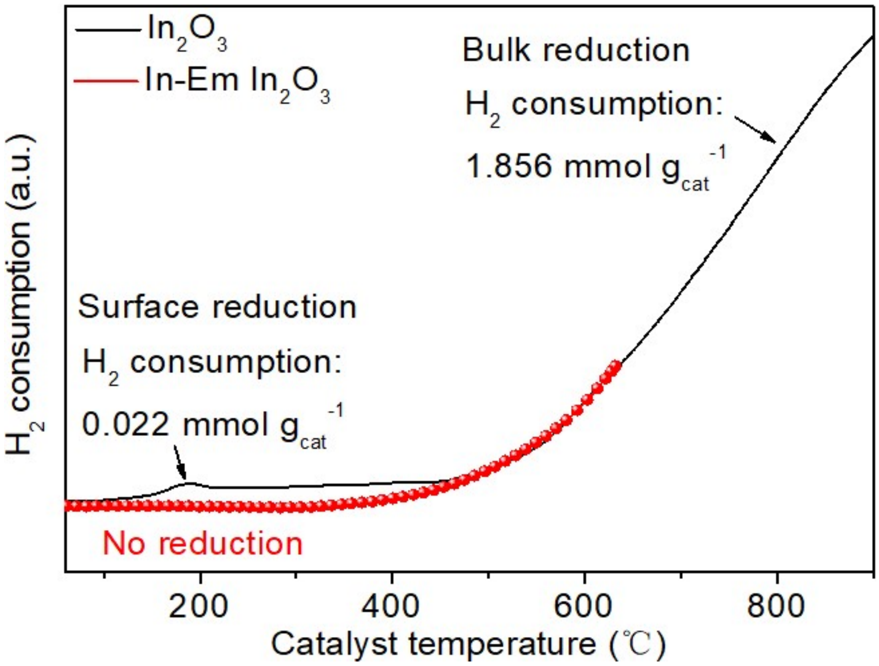

Supplementary Fig. 2 H_2-TPR patterns of In_2O_3 and In-Em In_2O_3.

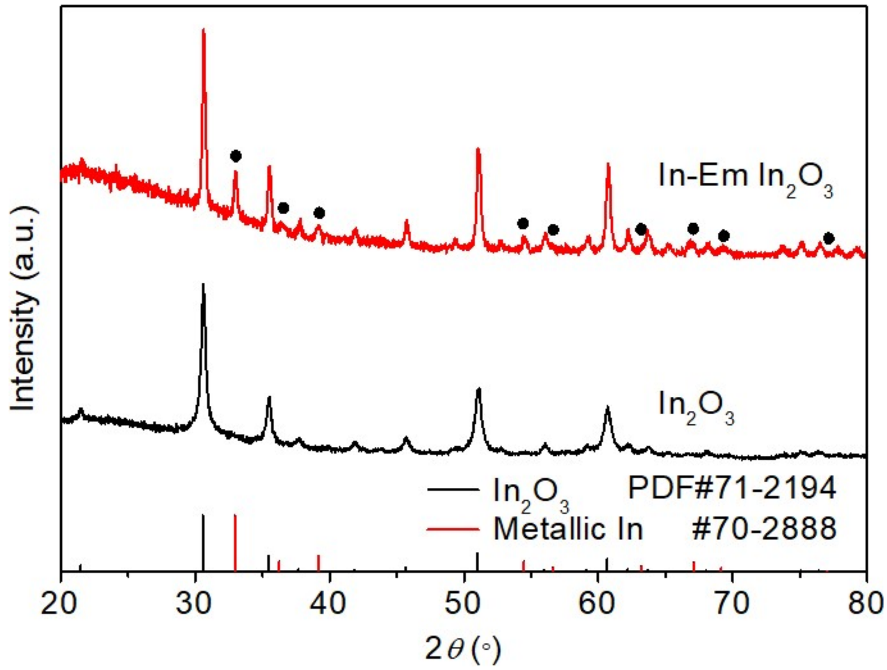

Supplementary Fig. 3 XRD patterns of In_2O_3 and In-Em In_2O_3.

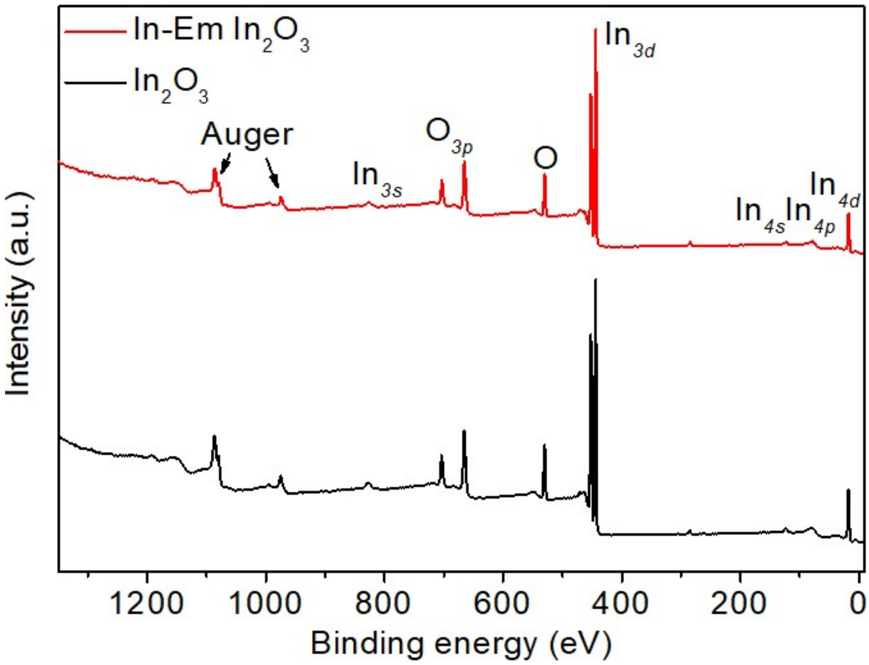

Supplementary Fig. 4 XPS survey spectra of In_2O_3 and In-Em In_2O_3.

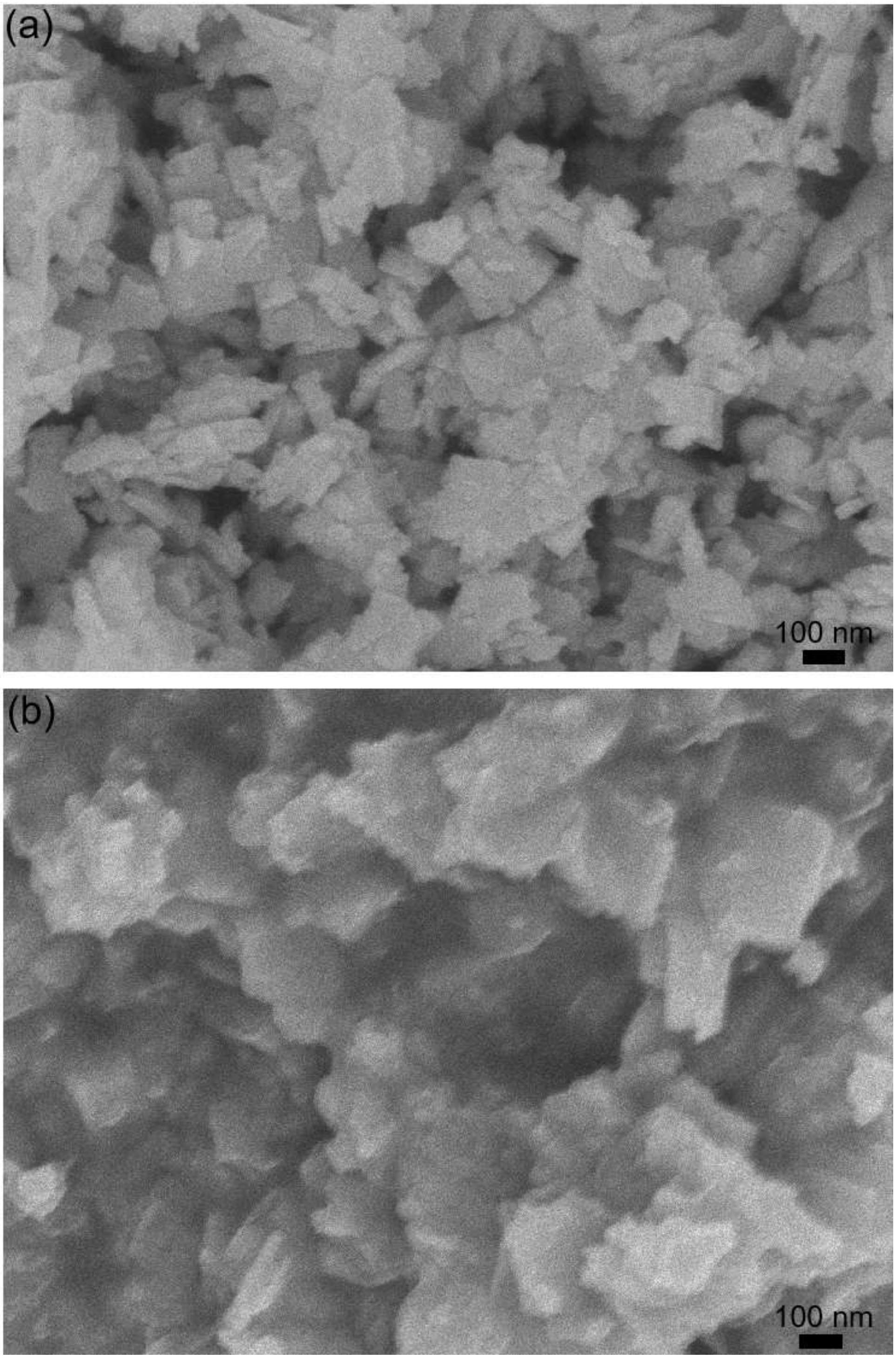

Supplementary Fig. 5 SEM images of **a** In_2O_3 and **b** In-Em In_2O_3.

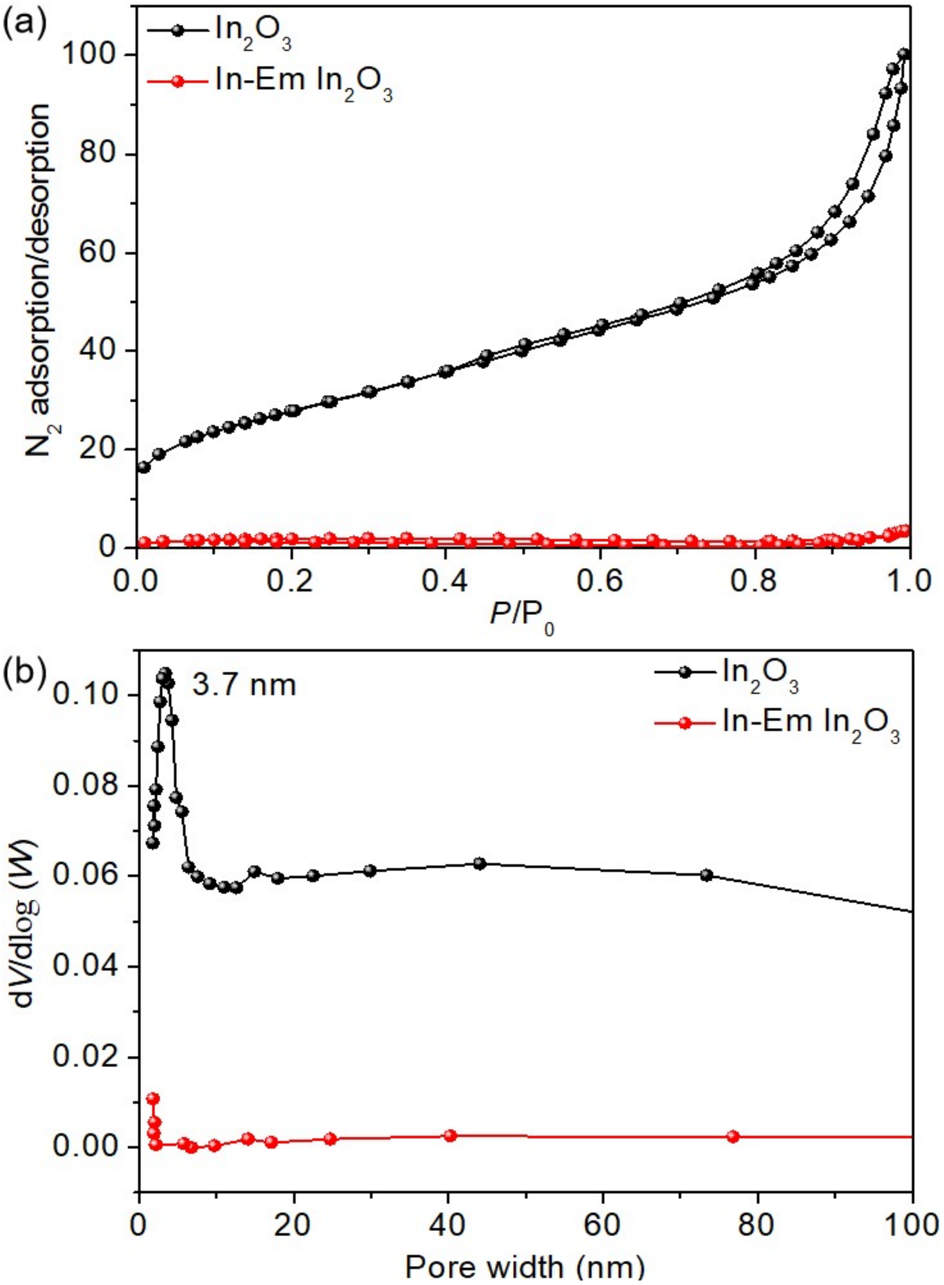

Supplementary Fig. 6 a N_2 adsorption-desorption isothermal curves of In_2O_3 and In-Em In_2O_3. The curves of In_2O_3 approximately correspond to type IV isotherm for typical mesoporous solid, while the curve of In-Em In_2O_3 approximately to type II isotherm for nonporous material. The type of hysteresis loops corresponds to type H3 due to the aggregates of plate-like particles. **b** Pore size distribution curves of In_2O_3 and In-Em In_2O_3. The pores of In_2O_3 are relatively obvious and the pore width corresponds to 3.7 nm, while there is no obvious pore size distribution for In-Em In_2O_3.

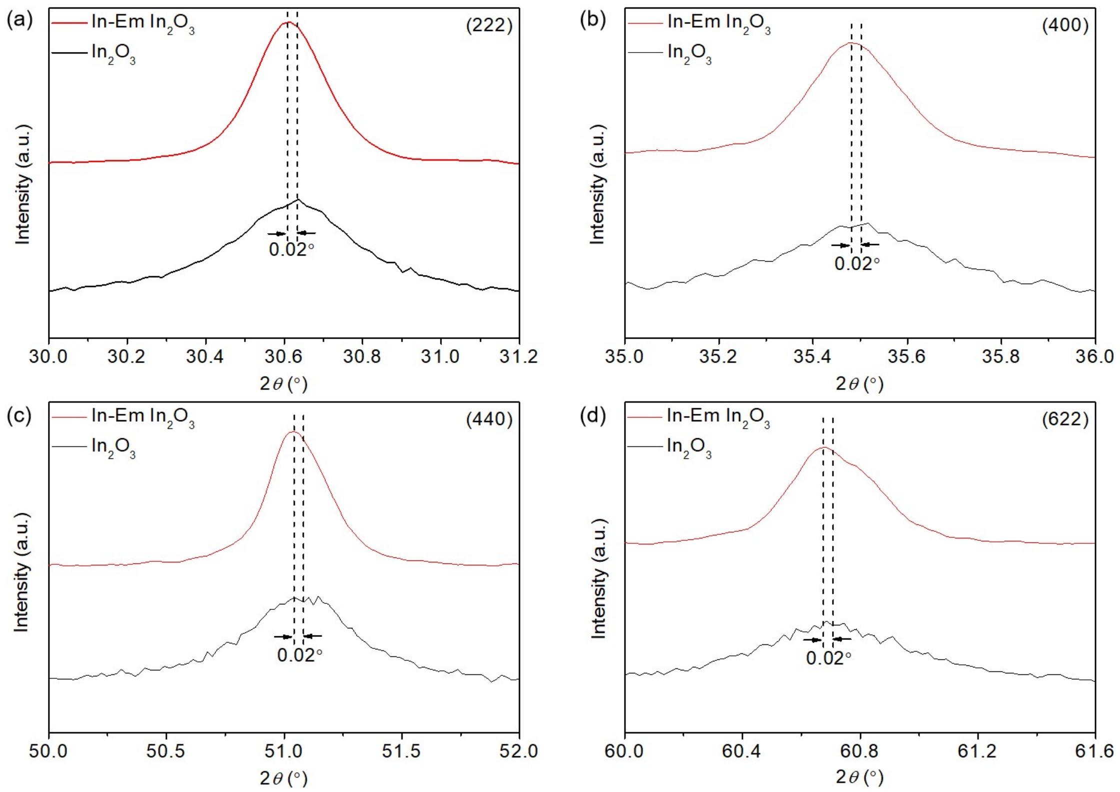

Supplementary Fig. 7 Magnified XRD patterns with the corresponding **a** facet (222), **b** facet (400), **c** facet (440) and **d** facet (622) of In_2O_3 and In-Em In_2O_3. The differences of their 2θ values are only 0.02º, which are one order of magnitude less than the reported XRD peak shift involving the strain effect induced by dopants[12]. In spite of decreased size, In-Em In_2O_3 exhibits the similar strain effect with In_2O_3 which is engendered by local disorder structure in the bulk, accounting for negligible XRD peak variation.

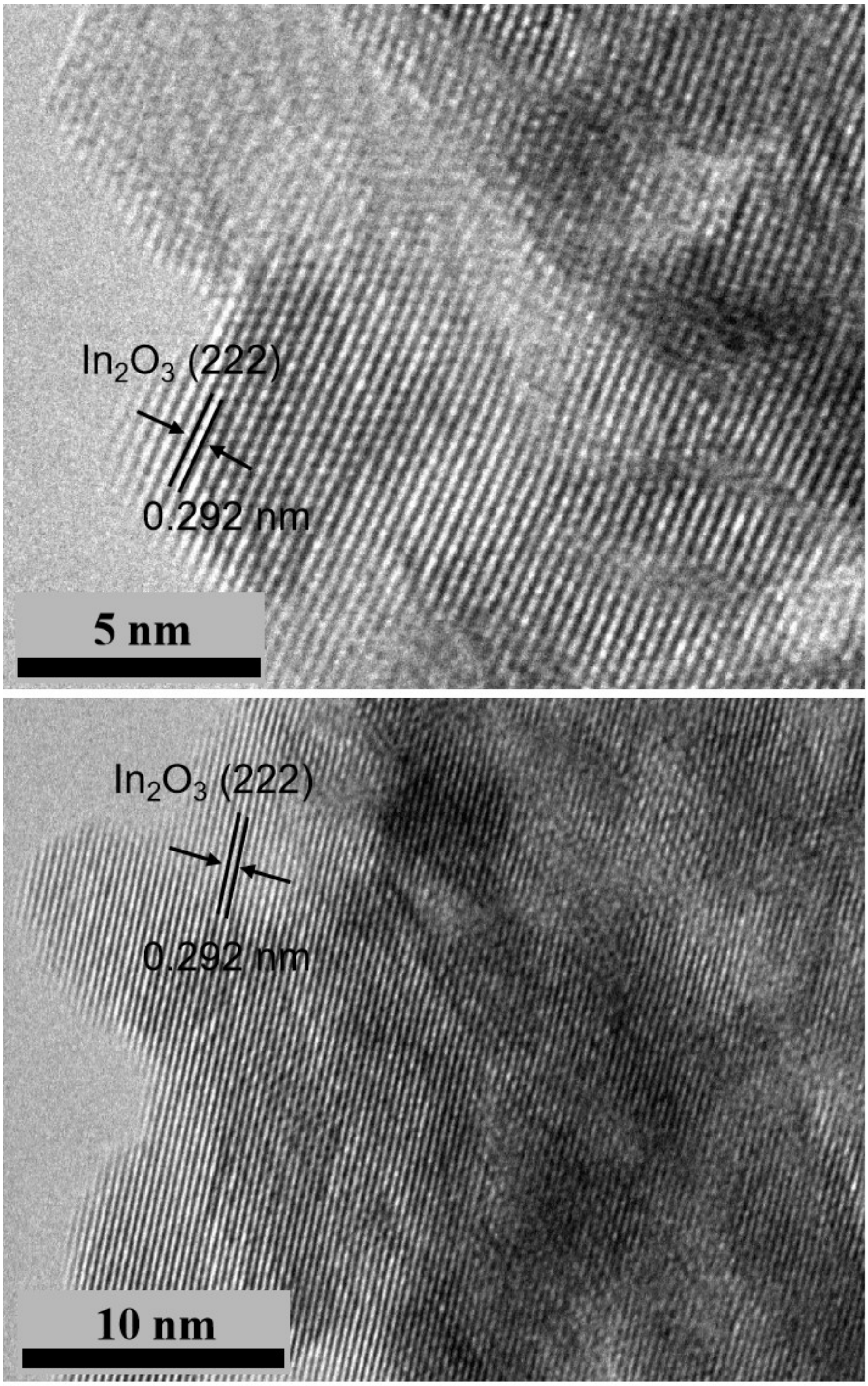

Supplementary Fig. 8 HRTEM images of In_2O_3. The lattice fringe with an interplanar spacing of 2.92 Å for In_2O_3 (222) facet is observed.

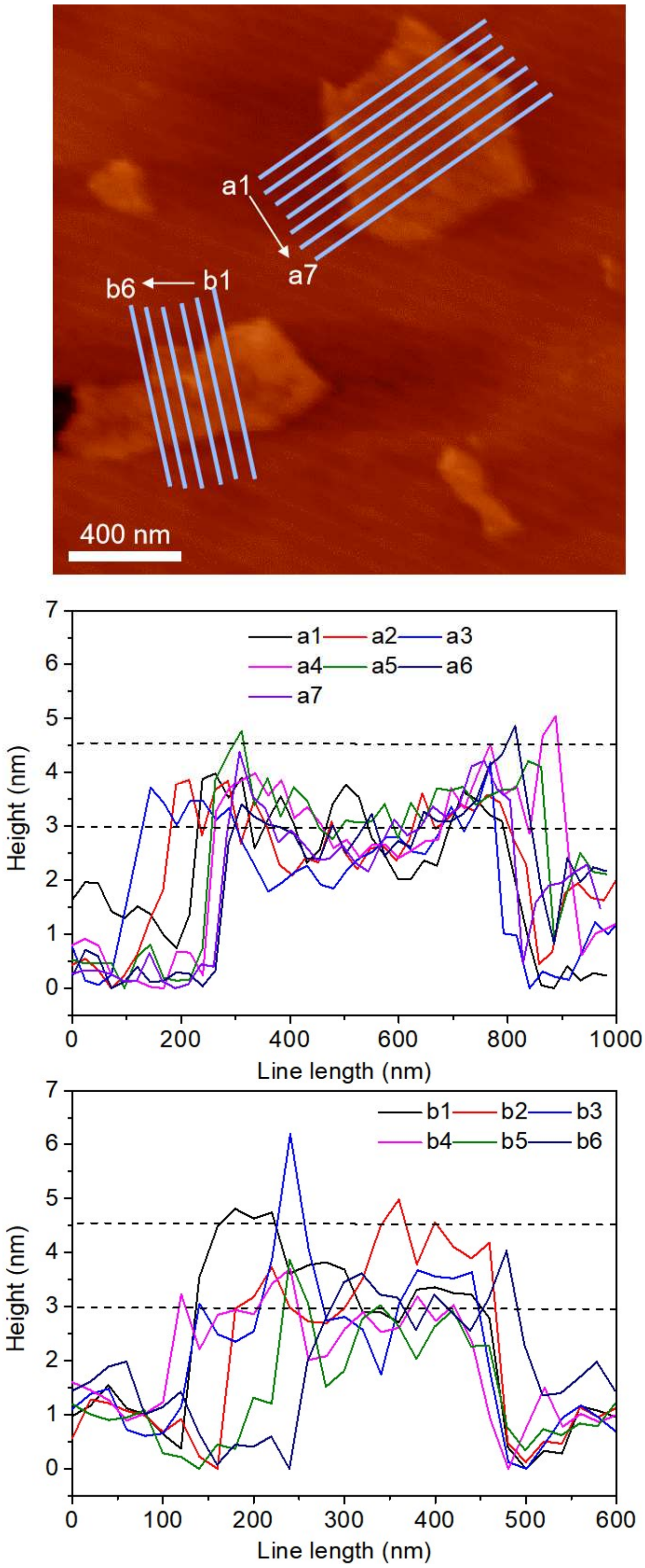

Supplementary Fig. 9 AFM image of In-Em In_2O_3 and the height along the lines. The AFM image presents an isolated nanoflake. To exclude accidental factors and make reasonable statistics, multiple straight lines on the surface were drawn. The undulate surface height is between 3 and 4.5 nm, reflecting the smooth surface of In-Em In_2O_3.

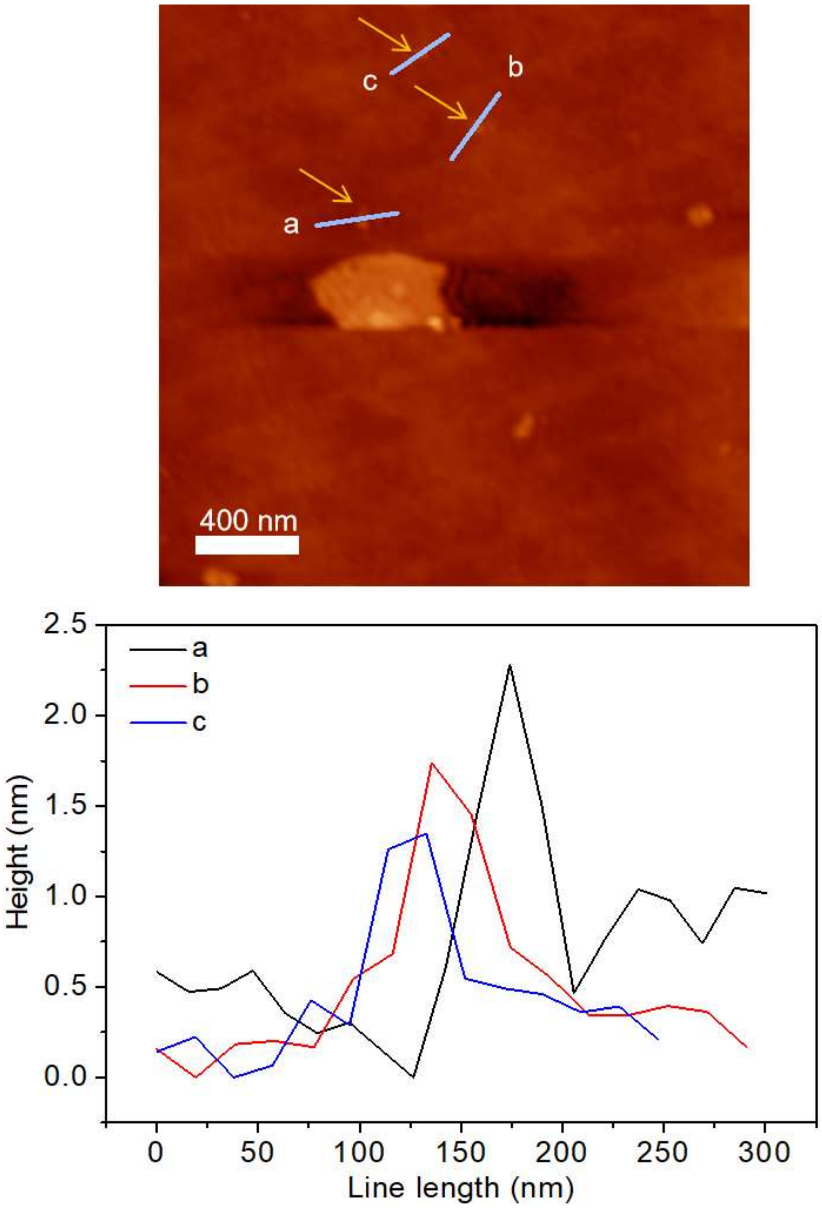

Supplementary Fig. 10 AFM image. There are some separated In nanoparticles (arrows) with the height of 1.5~2.5 nm.

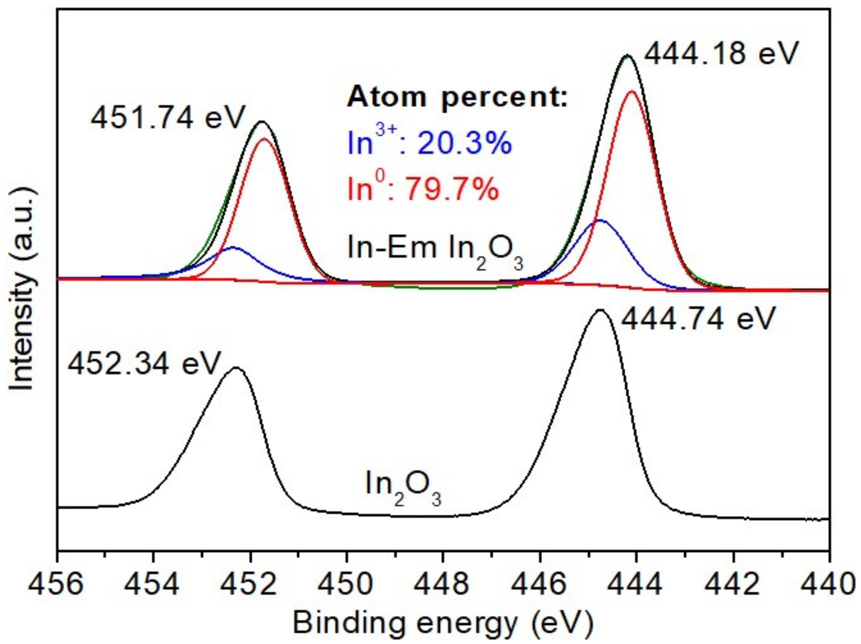

Supplementary Fig. 11 In_{3d} XPS spectra of In_2O_3 and In-Em In_2O_3.

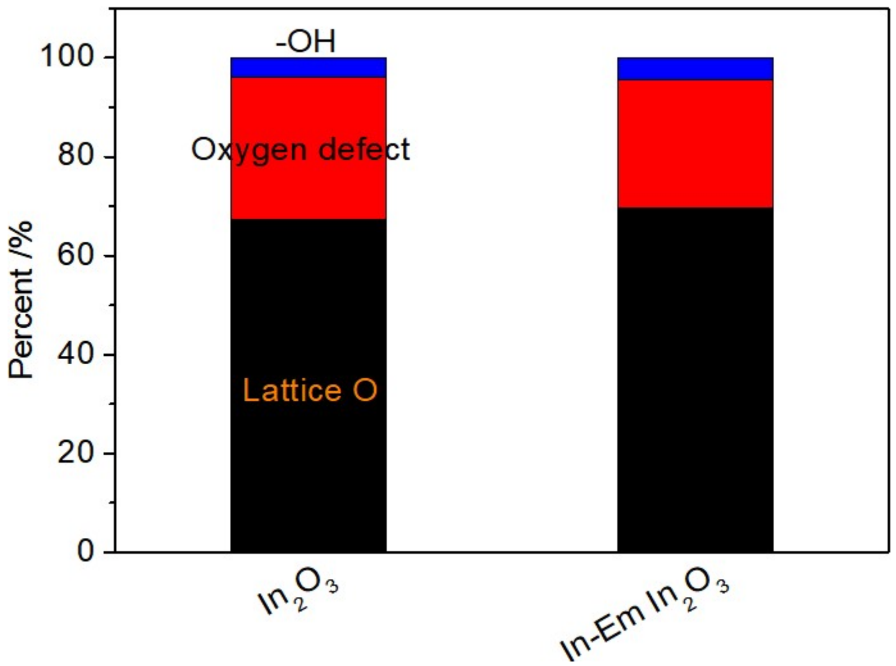

Supplementary Fig. 12 A histogram summarizing the oxygen species distribution in In_2O_3 and In-Em In_2O_3 from XPS spectra.

Supplementary Discussion: To eliminate the influence of crystal size on the calculation of oxygen concentration, we normalized oxygen concentrations by the specific surface area of single grain[7]. The grain size can be estimated via XRD patterns ((Supplementary Table 2) and then through

$$S=6/\rho*D \quad (16)$$

where S is specific surface area of grain, ρ is the density of In_2O_3 and D is grain size, we obtained the specific surface area of grain. Obviously, the specific surface areas of grains of In_2O_3 and In-Em In_2O_3 are nearly the same. Therefore, the oxygen-defect concentrations normalized by specific surface area of grain for the two catalysts are virtually similar.

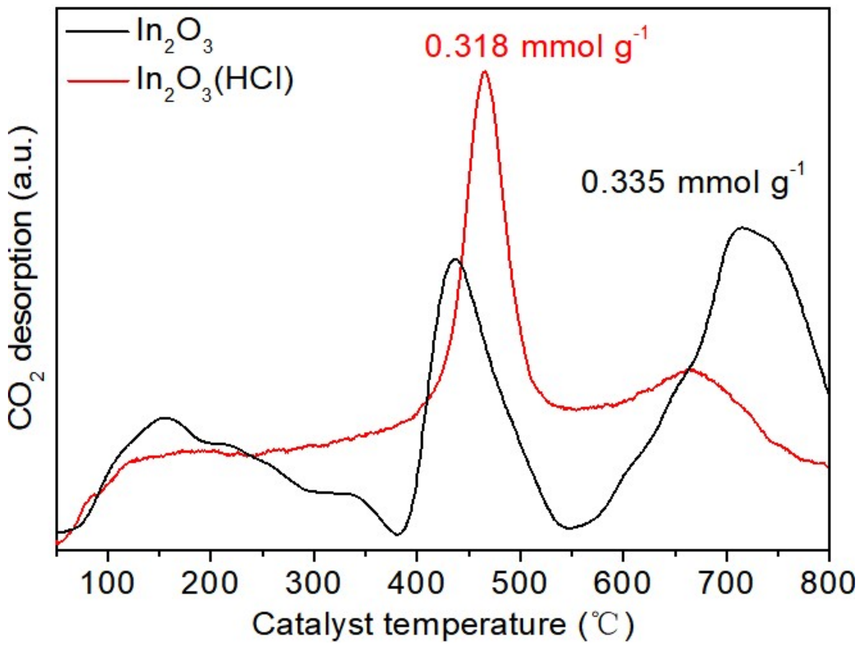

Supplementary Fig. 13 CO_2-TPD profiles of In_2O_3 and In_2O_3 with HCl etching. With HCl etching, CO_2 chemisorption strength changes likely because of surface modification by Cl atoms, but their CO_2 adsorption amount remains unchanged.

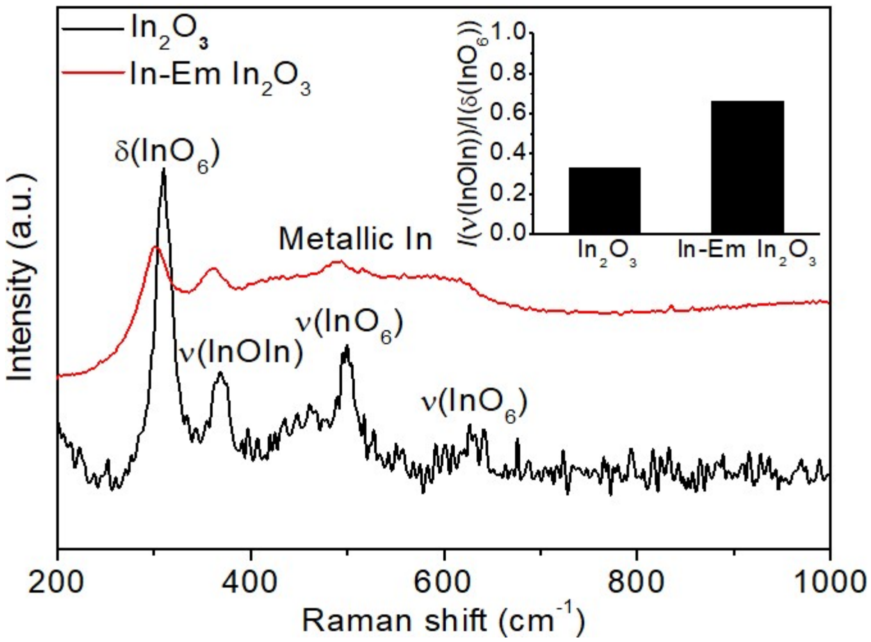

Supplementary Fig. 14 Raman spectra of In_2O_3 and In-Em In_2O_3. Inset: the intensity ratio of ν(InOIn) vs. δ(InO_6).

Supplementary Discussion: Raman spectra were used to analyze the bonding modes of the catalysts. The peaks at 309.4 and 500.3 cm^{-1} correspond to the In-O symmetric δ(InO_6) and antisymmetric ν(InO_6) stretching vibrations, respectively, while that at 365.9 cm^{-1} is attributed to the In-O-In structure[13-14]. The broad band at 400~700 nm is a typical feature of metal[15] for In-Em In_2O_3. Compared with In_2O_3, the red shift of all the peaks for In-Em In_2O_3 suggests that there is a weaker In-O binding and this accords well with the results from the O_{1s} XPS spectra. Importantly, the proportion of In-O-In of In-Em In_2O_3 is higher compared with In_2O_3; therefore, In-O-In can be implicated as the basic structural unit of the oxygen defects of In-Em In_2O_3.

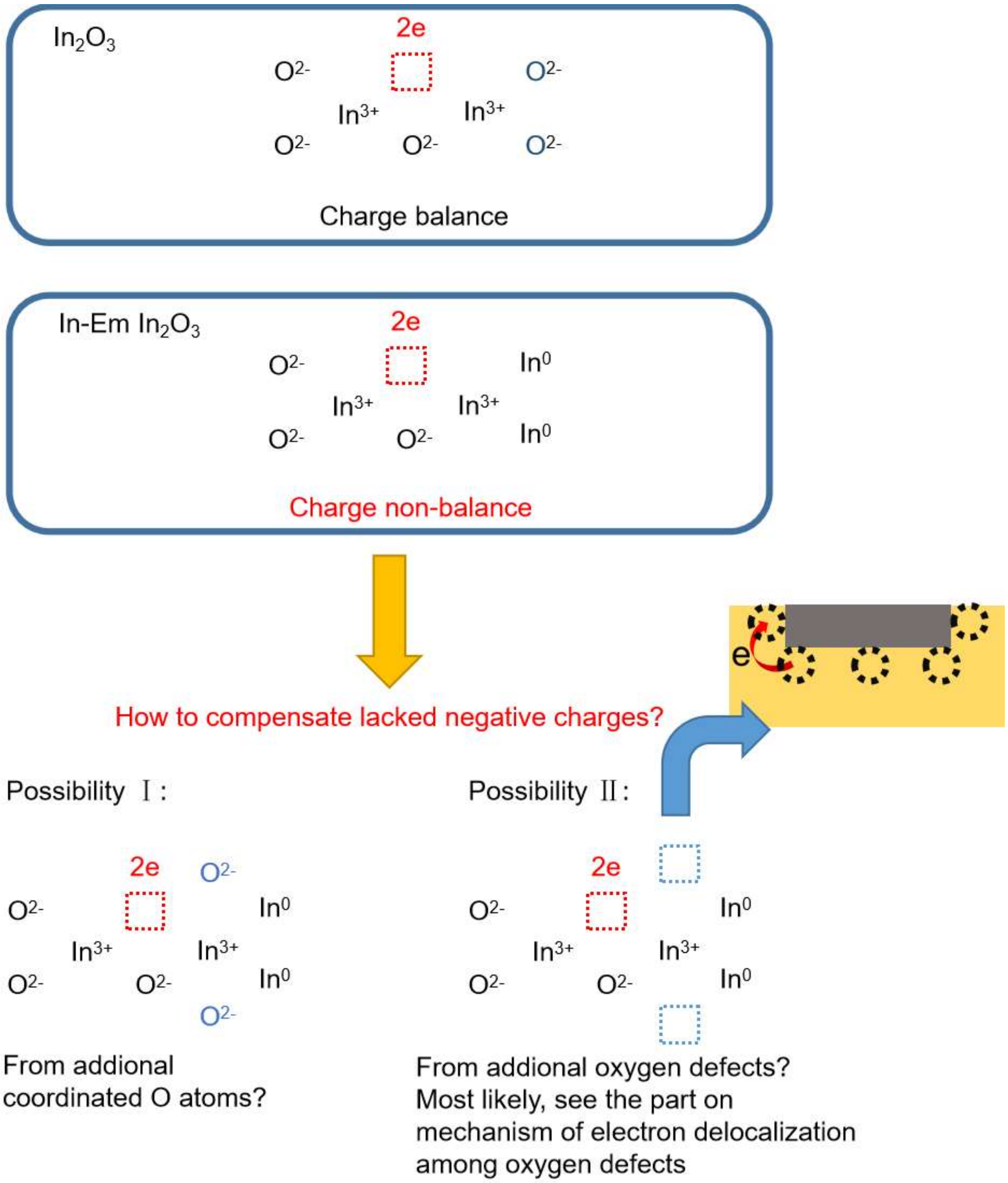

Supplementary Fig. 15 Analysis of charge balance around oxygen defects.

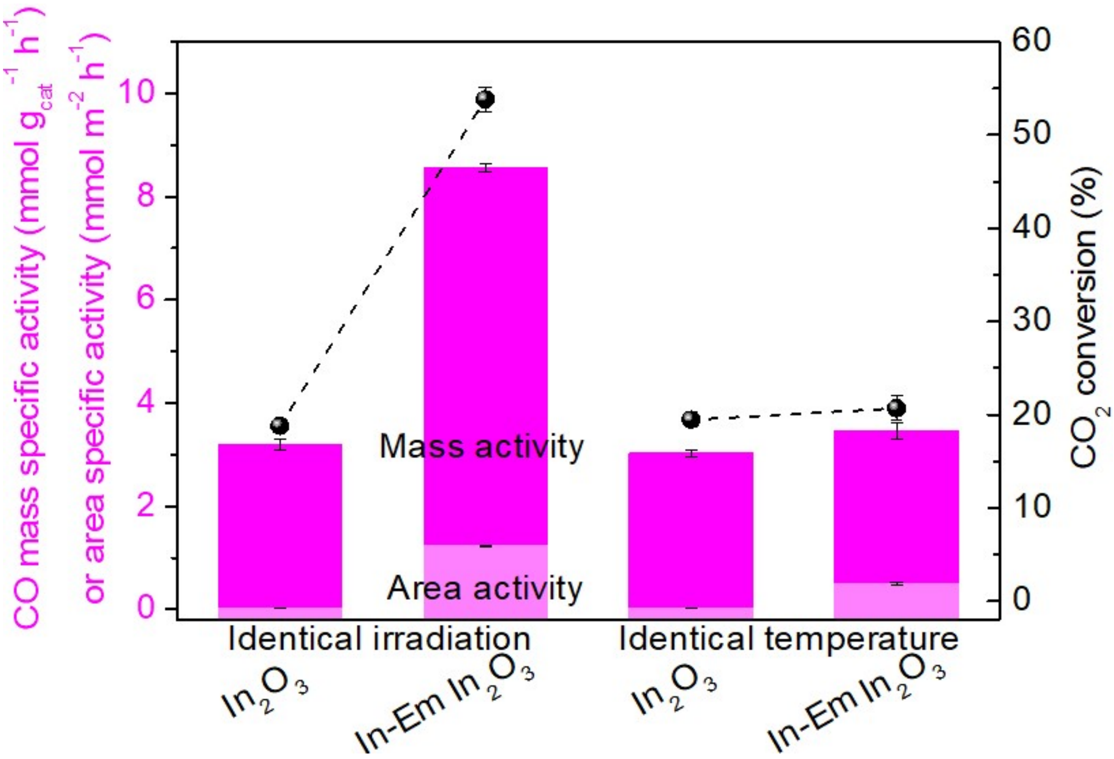

Supplementary Fig. 16 CO mass/area specific activities and CO_2 conversions over In_2O_3 and In-Em In_2O_3 at identical full-spectrum light intensity and reaction temperature of 300℃. (The error bars represent standard deviation.)

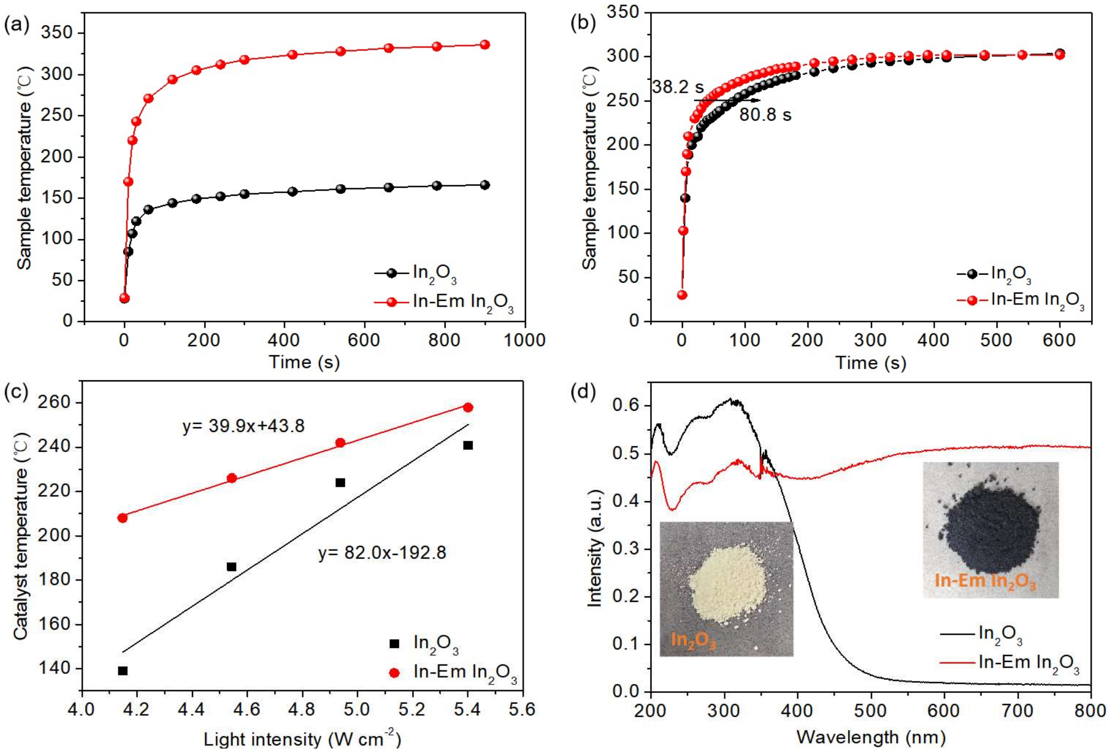

Supplementary Fig. 17 **a** Temperature profiles for reactor with In_2O_3 and In-Em In_2O_3 under identical full-spectrum light irradiation. **b** Temperature profiles via controlling final temperature to 300℃. **c** Different light intensity corresponds to different catalyst temperature. **d** Absorption spectra.

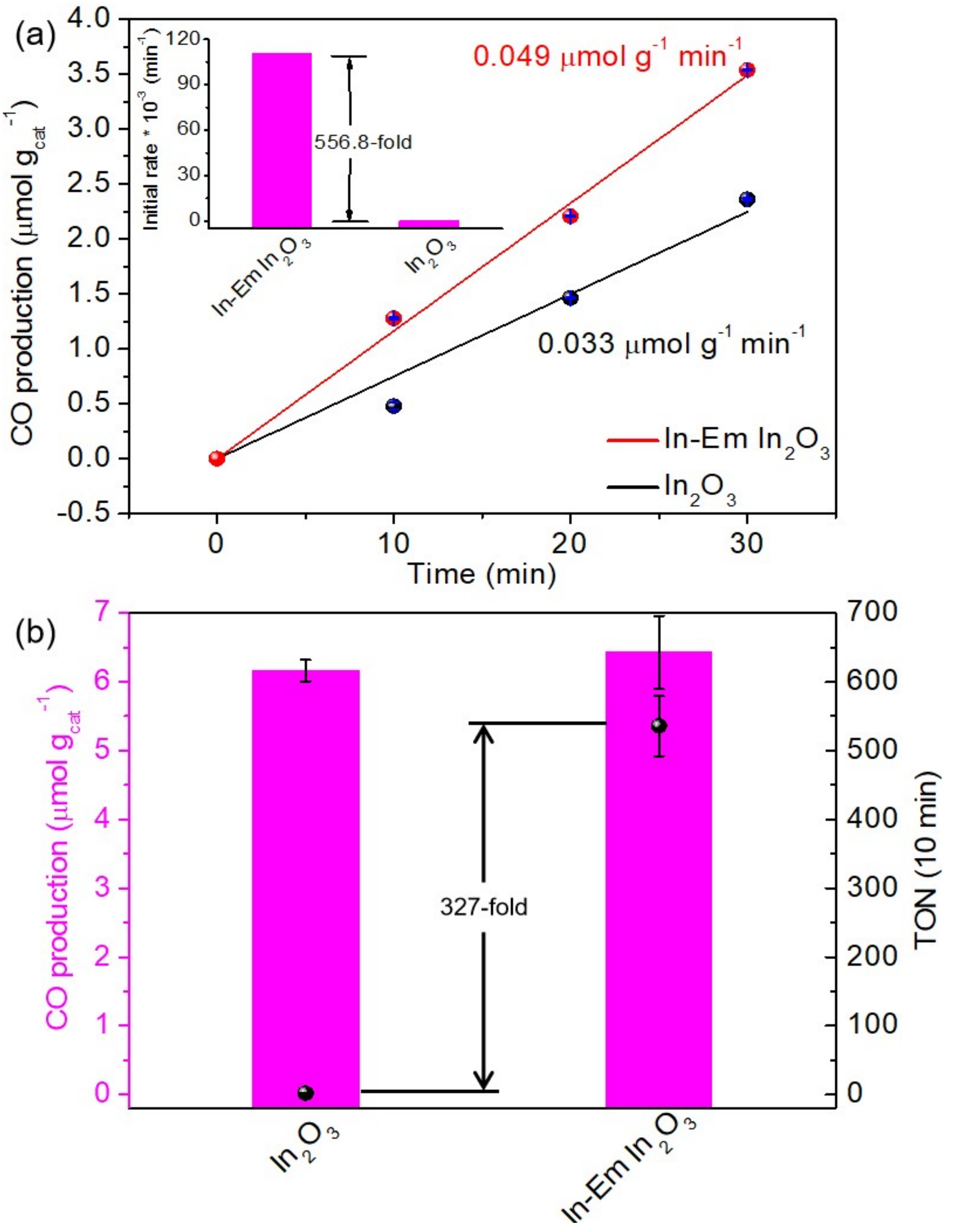

Supplementary Fig. 18 Maintenance of oxygen defects. **a** At the reaction temperature of 250℃, reaction time dependence of CO productions (Inset: initial reaction rates normalized by number of active sites). **b** At the reaction temperature of 350℃, CO productions and TONs over 10 min. (The error bars represent standard deviation.)

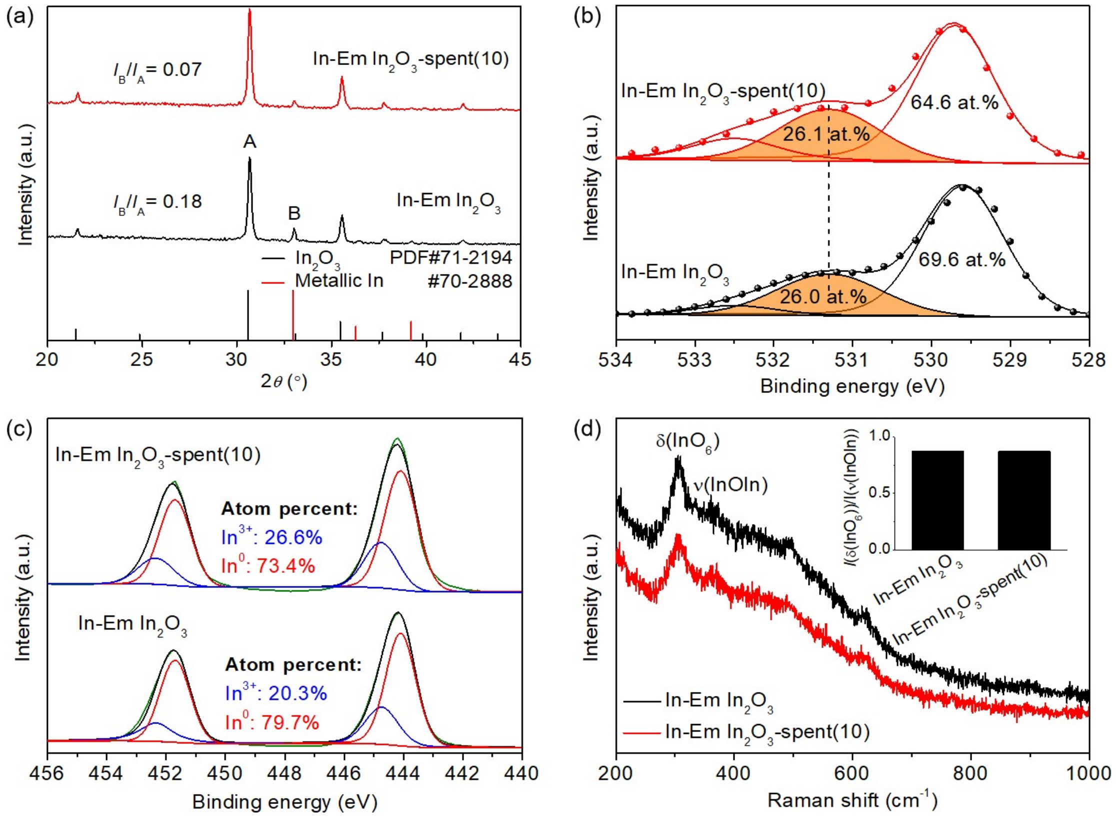

Supplementary Fig. 19 a XRD patterns, **b** O_{1s} XPS spectra, **c** In_{3d} XPS spectra, **d** Raman spectra of In-Em In_2O_3 and In-Em In_2O_3-spent(10) where “10” refers to that the catalyst underwent ten runs of reaction.

.

Supplementary Discussion: The structure, composition of the catalyst spent was identified to explain the nature of catalytic stability. First, as demonstrated in XRD patterns (Supplementary Fig. 19a), after the photothermocatalytic reaction, the content of metallic In of In-Em In_2O_3 was decreased because of the oxidation of the metallic In by the OH intermediate formed via the dissociation of COOH. Both the embedded and supported metallic In would be oxidized, however, this change can not affect the activity of the catalyst. Second, XPS spectra were used to analyze the surface component of In-Em In_2O_3-spent. The atom percent of oxygen defects remains (Supplementary Fig. 19b). Moreover, the content of In^0 on the surface of the catalyst spent was slightly decreased by 6.3% (Supplementary Fig. 19c). Therefore, it is most likely to deduce that the structure and content of oxygen defects on the (sub)surface were retained during the photothermocatalytic reaction. TPD patterns were used to analyze the amount of the outermost layer of surface oxygen defects. The results were summarized in Supplementary Table 5. At one run of reaction, the amount of CO_2 adsorbed was unchanged, indicating the similar content of surface oxygen defects, which can be used to explain the unchanged performance. After ten runs of reaction, though the amount of surface oxygen defects increases by a factor of 40, it is much lower related to In_2O_3 (0.335 mmol g^{-1}), suggesting that the higher reactivity of such oxygen defects is still dominant. That seems that the content and reactivity of oxygen defects complement each other for stable performance. Raman spectra demonstrate the same ratio of ν(InOIn) vs. δ(InO_6) (Supplementary Fig. 19d), indicating the unchanged interaction mode between In_2O_3 and metallic In and similar concentration of oxygen defect complex (O-In-(O)Vo-In-In structure).

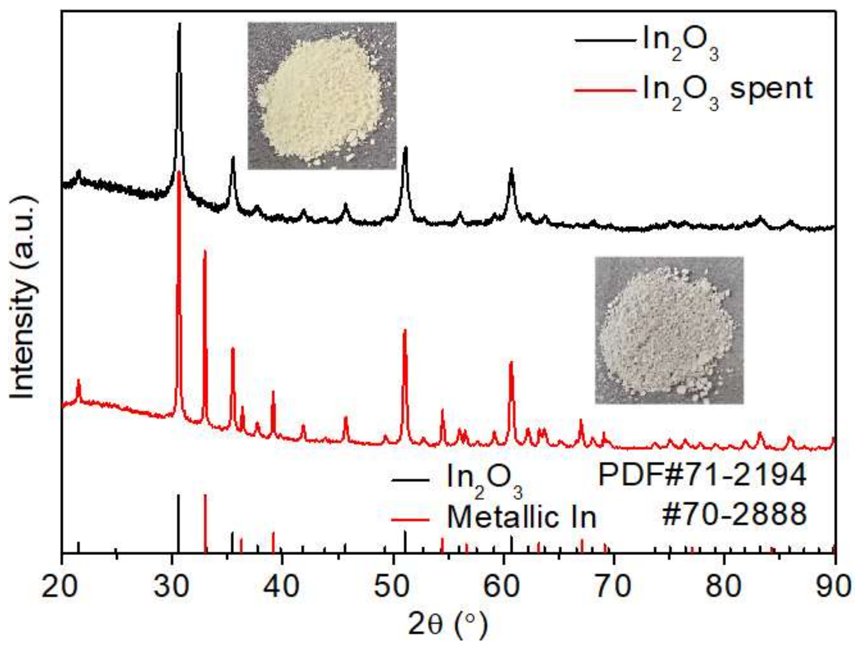

Supplementary Fig. 20 XRD patterns of In_2O_3 and In_2O_3 spent. Here the reaction was executed at the temperature of 350°C. The color changed from light yellow to gray.

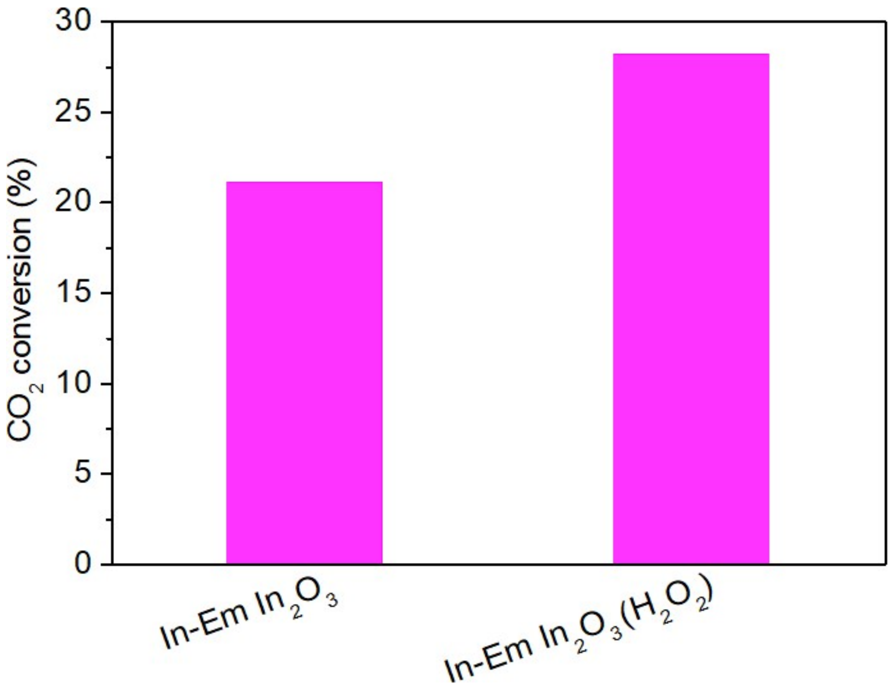

Supplementary Fig. 21 Performance comparison over In-Em In_2O_3 and In-Em $In_2O_3(H_2O_2)$.

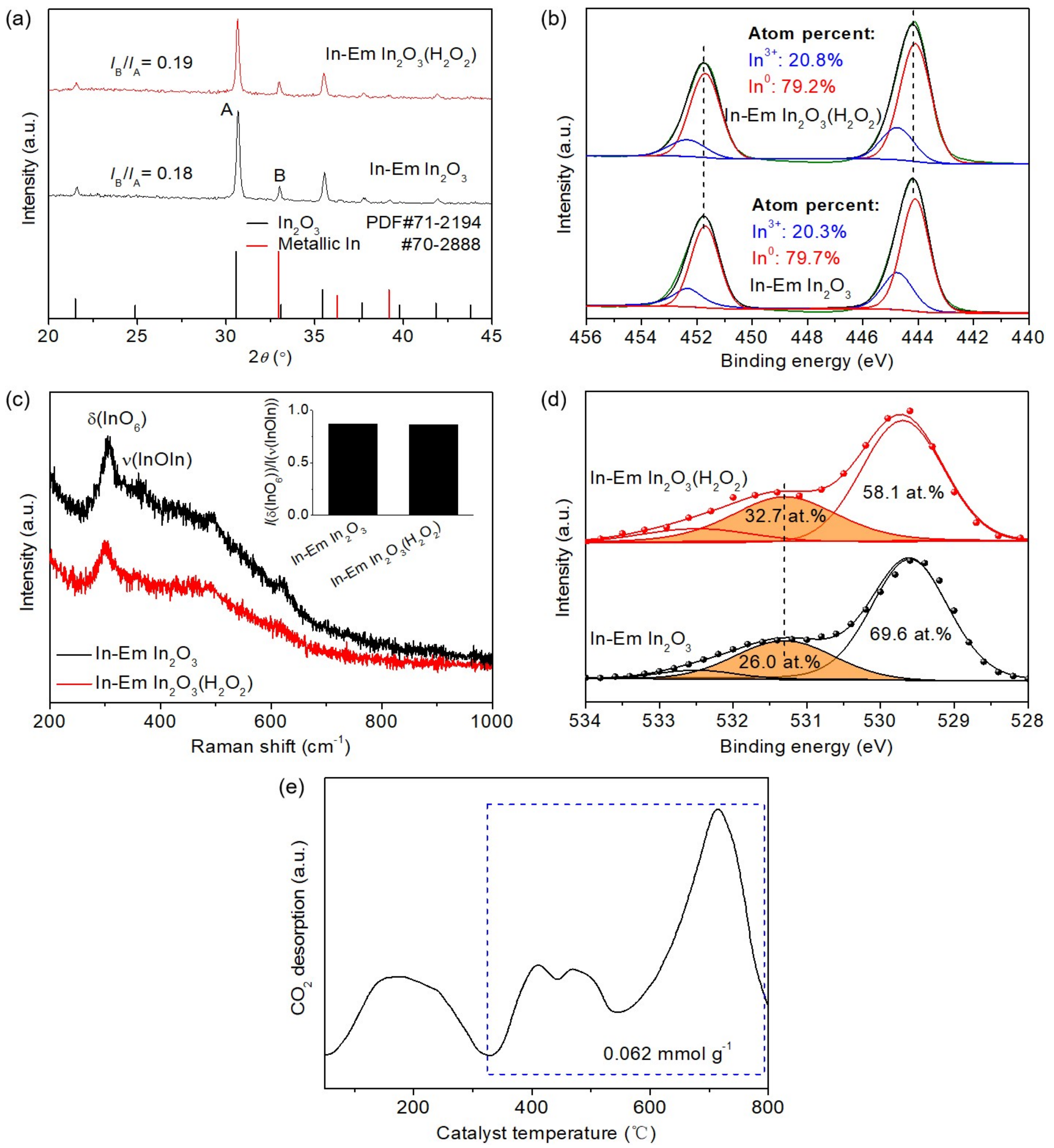

Supplementary Fig. 22 a XRD patterns, **b** In_{3d} XPS spectra, **c** Raman spectra, **d** O_{1s} XPS spectra and (e) CO_2-TPD patterns of In-Em $In_2O_3(H_2O_2)$.

Supplementary Discussion: It can be seen from XRD patterns (Supplementary Fig. 22a) that the diffraction peak intensity ratio of metallic In/In_2O_3 does not change, indicating that the hydrogen peroxide treatment does not change the overall ratio of metallic In/In_2O_3. Moreover, In_{3d} XPS spectra show that the surface component of In species change slightly (Supplementary Fig. 22b). Also, the interaction mode between In_2O_3 and metallic In and concentration of oxygen defect complex (O-In-(O)Vo-In-In structure) remain as indicated by Raman spectra (Supplementary Fig. 22c). It can be surmised that the metallic In on the surface was oxidized, forming a very thin layer of indium oxide which possesses a certain amount of surface oxygen defects. Indeed, the O_{1s} XPS spectra show that the content of surface oxygen defects was increased by 6.7% (Supplementary Fig. 22d). CO_2-TPD patterns verify that the increased oxygen defects are on the outermost surface (Supplementary Fig. 22e). This further confirms the accuracy of our conclusion that the oxygen defects around metallic In exhibit the higher reactivity.

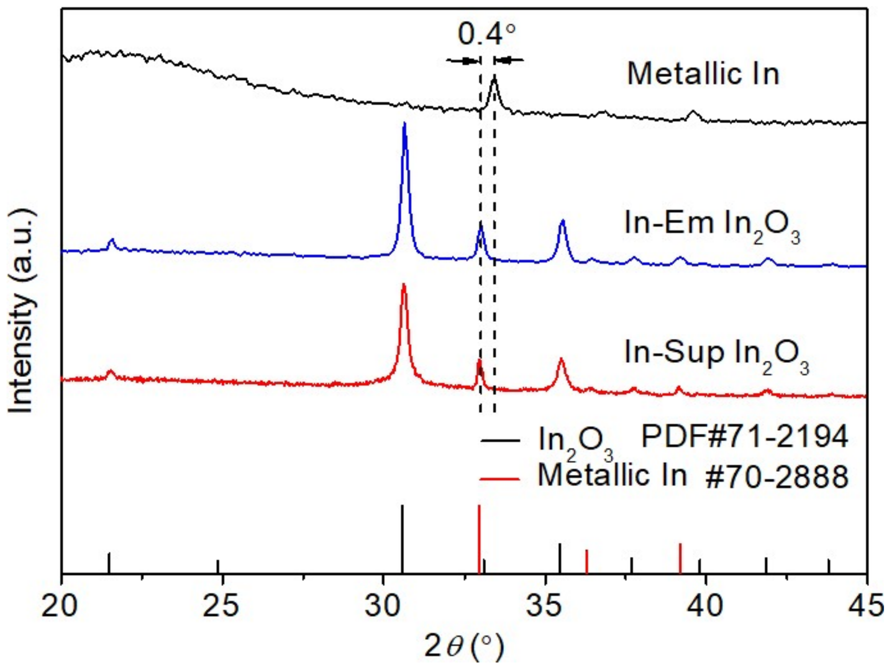

Supplementary Fig. 23 XRD patterns of metallic In, In-Em In_2O_3 and In-Sup In_2O_3.

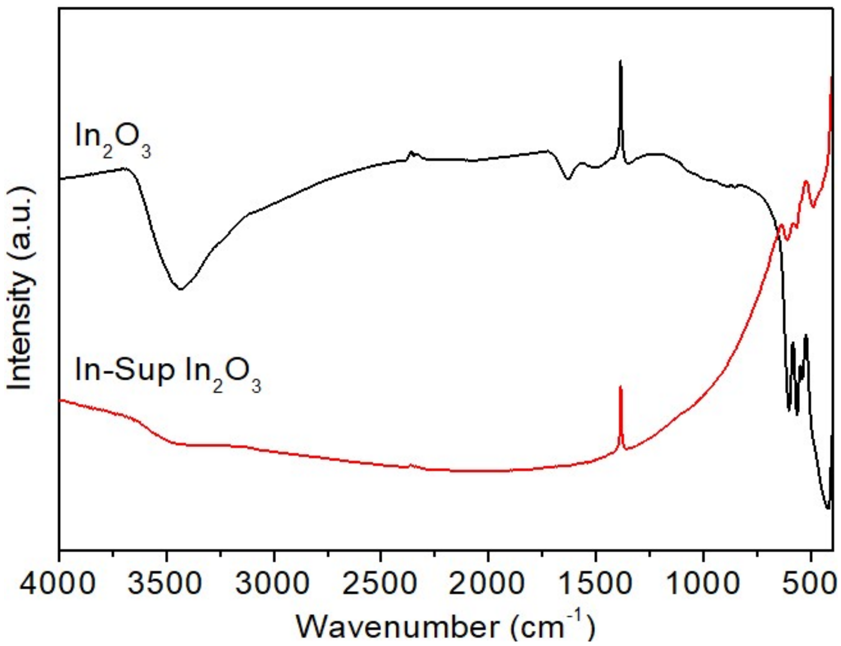

Supplementary Fig. 24 FT-IR spectra of In_2O_3 and In-Sup In_2O_3.

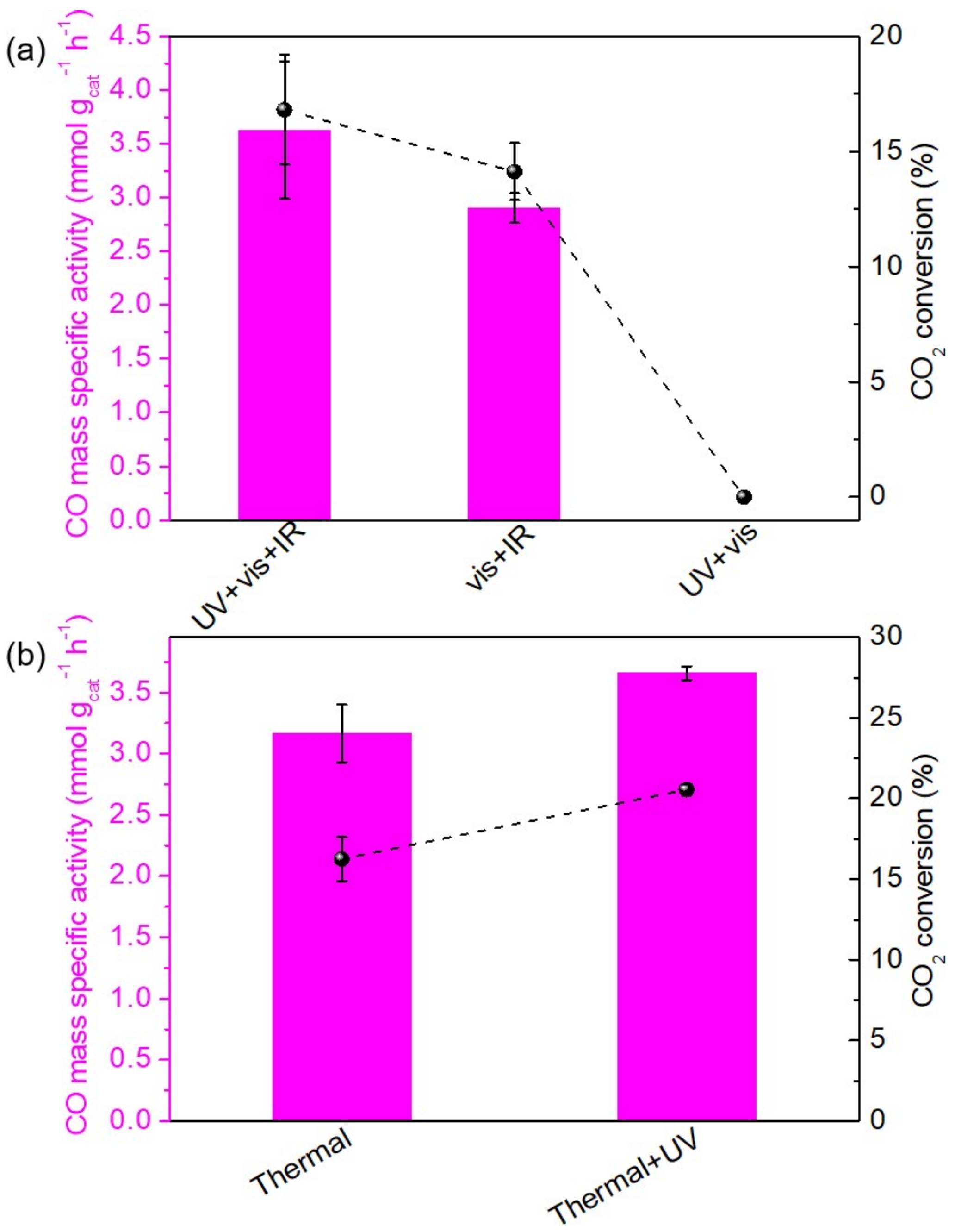

Supplementary Fig. 25 a Photothermal catalytic performances under UV+vis+IR and vis+IR light irradiation and photocatalytic performance (190 nm< λ< 760 nm). **b** Performance comparison between thermal and thermal+UV (2.9 W cm^{-2}) catalysis at 350℃. (The error bars represent standard deviation.)

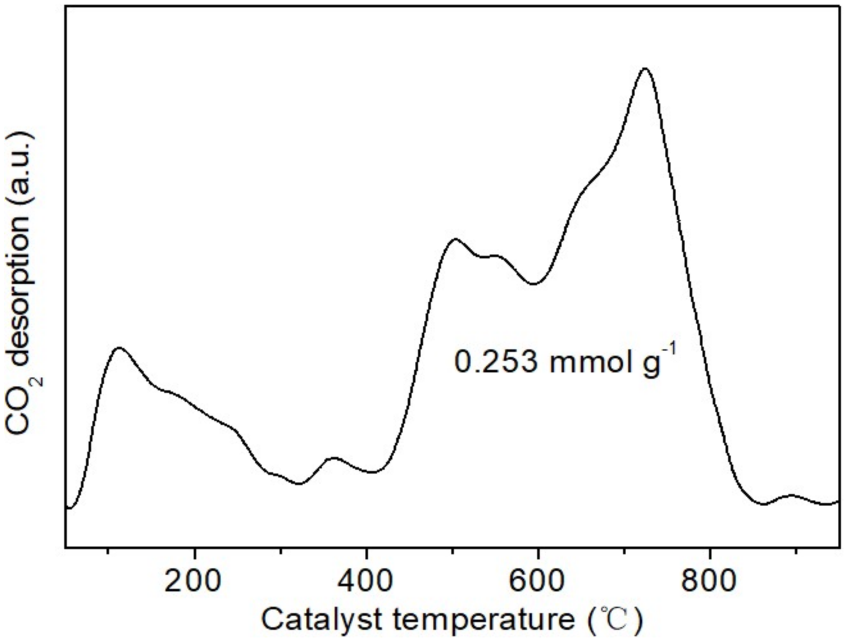

Supplementary Fig. 26 CO_2-TPD profile of In-Sup In_2O_3.

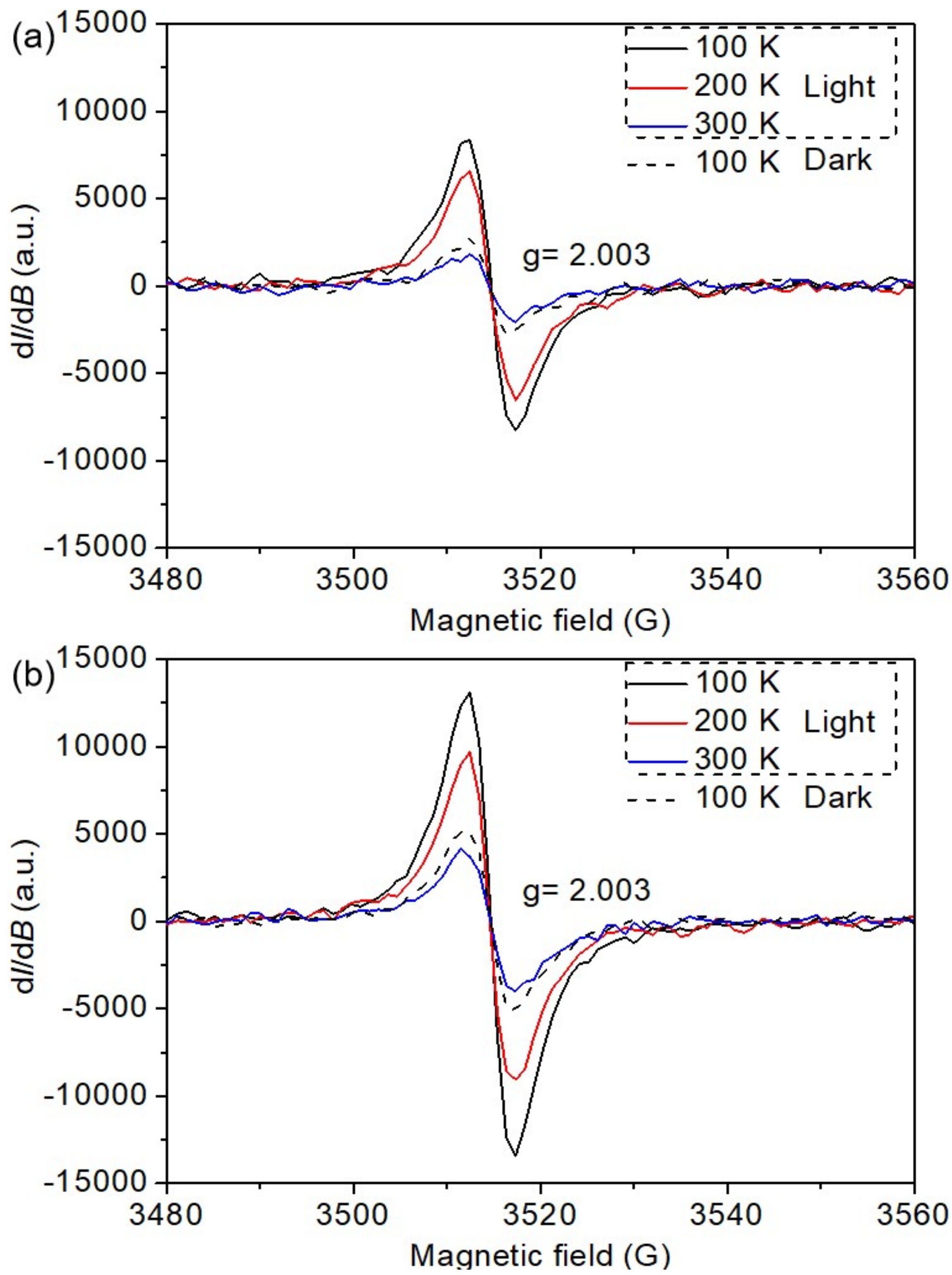

Supplementary Fig. 27 Variable temperature ESR spectra of **a** In_2O_3 and **b** In-Em In_2O_3 at 100, 200 and 300 K. The solid line and dashed line represent light irradiation signal and dark signal, respectively. An isotropic ESR singlet at g= 2.003 results from the allowed transitions $\Delta m_S = \pm 1$ at the same resonant field, suggesting that the coordination of oxygen defect site is highly symmetric and there is no zero-field splitting.

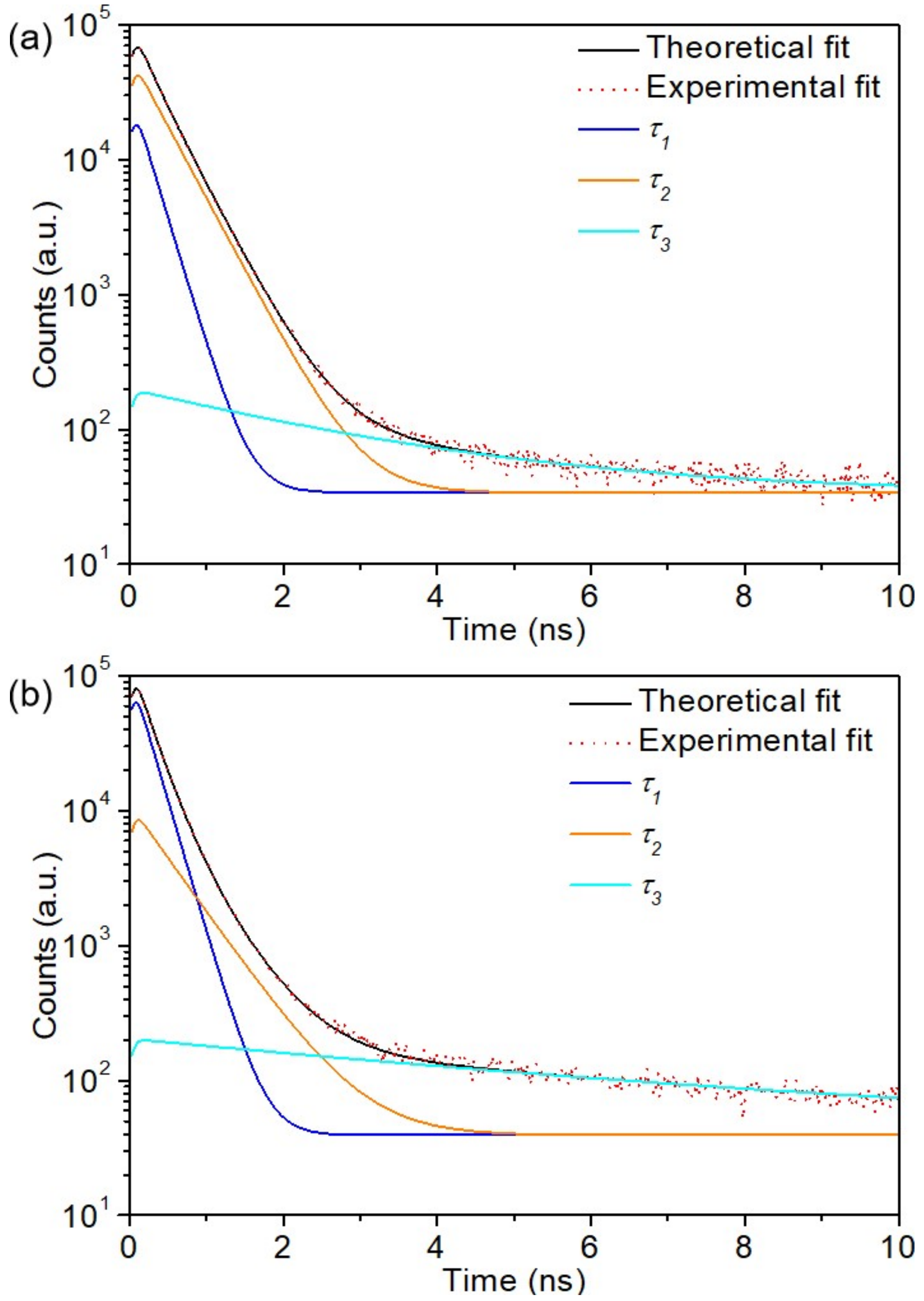

Supplementary Fig. 28 Positron annihilation discrete spectra of **a** In_2O_3 and **b** In-Em In_2O_3, including the corresponding discrete spectra of three lifetime components. The total numbers of annihilated positrons over In_2O_3 and In-Em In_2O_3 are the same, hence the relative intensities are equivalent to the concentration of annihilated positrons for calculating the positron annihilation rate constants over In_2O_3 and In-Em In_2O_3.

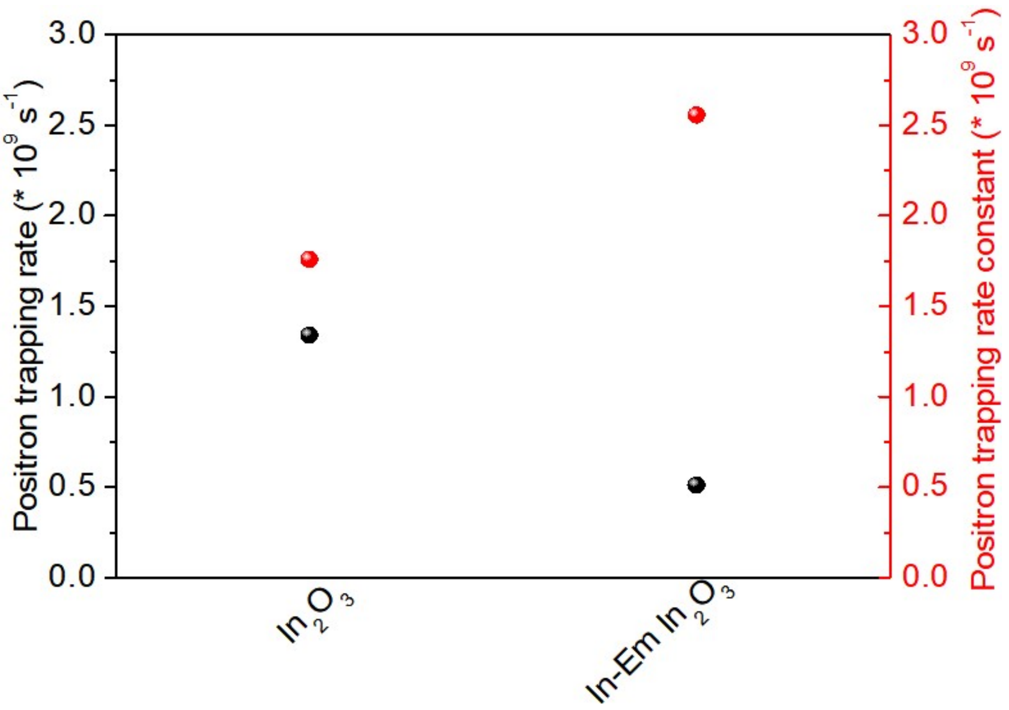

Supplementary Fig. 29 Positron trapping rates and trapping rate constants over In_2O_3 and In-Em In_2O_3.

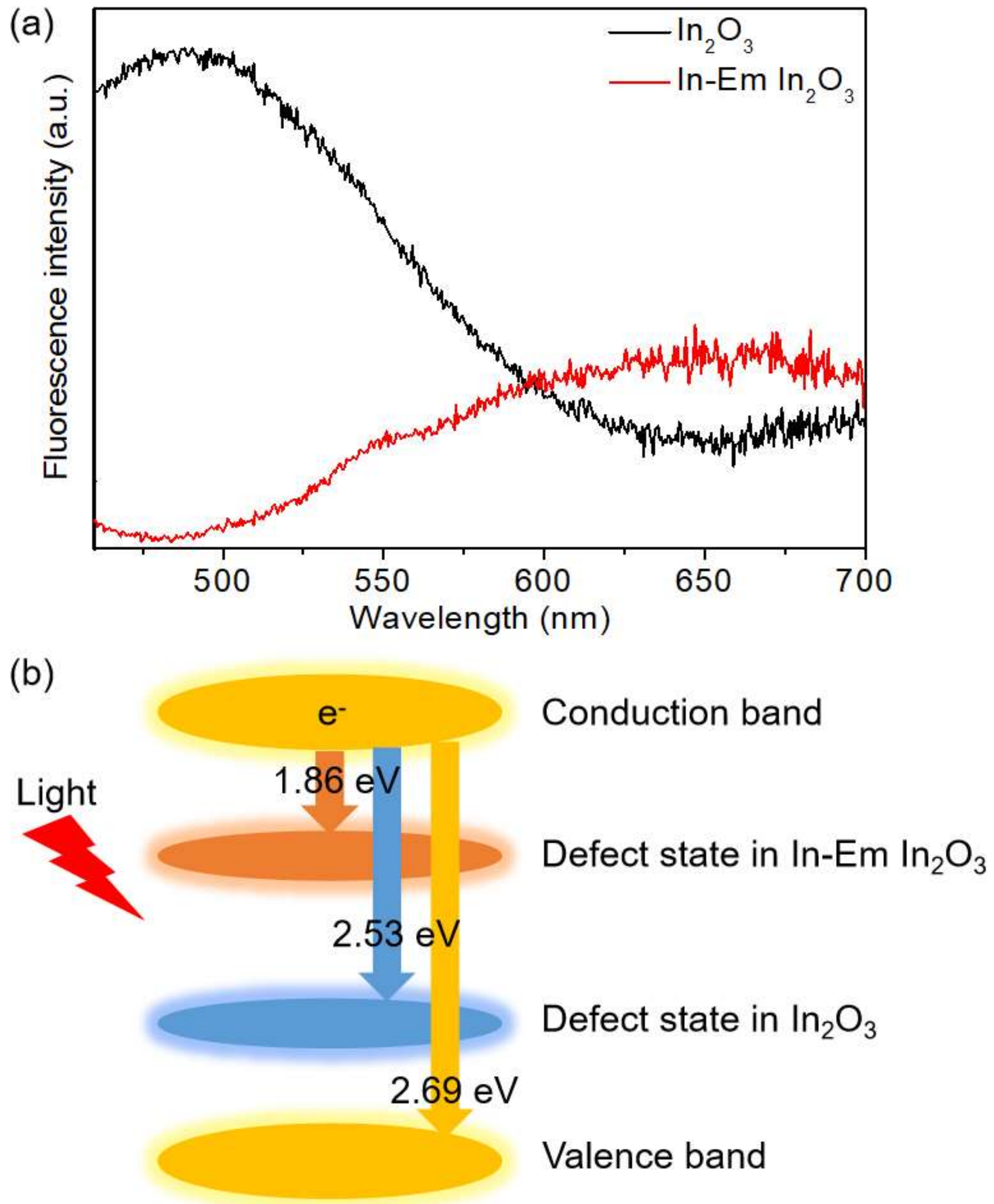

Supplementary Fig. 30 a Fluorescence spectra corresponding to the oxygen defects of In_2O_3 and In-Em In_2O_3 with fluorescence energy as X axis. **b** Energy levels of defect state in In_2O_3 and In-Em In_2O_3.

Supplementary Discussion: The fluorescence emission intensity of In-Em In_2O_3 corresponding to the oxygen defects is significantly weaker than that of In_2O_3, since the oxygen defects electronically interact with metallic In. Moreover, the emission wavelength of In-Em In_2O_3 moves to a lower energy compared to In_2O_3. Excitation energies of electrons in the oxygen defects of In_2O_3 and In-Em In_2O_3 correspond to 2.53 and 1.86 eV, respectively. Given that the conduction-band potentials are the same, the energy levels of the defect states are compared: In-Em In_2O_3> In_2O_3, thus electrons in the oxygen defects of In-Em In_2O_3 have more potential to get rid of some electrostatic field around the oxygen defects.

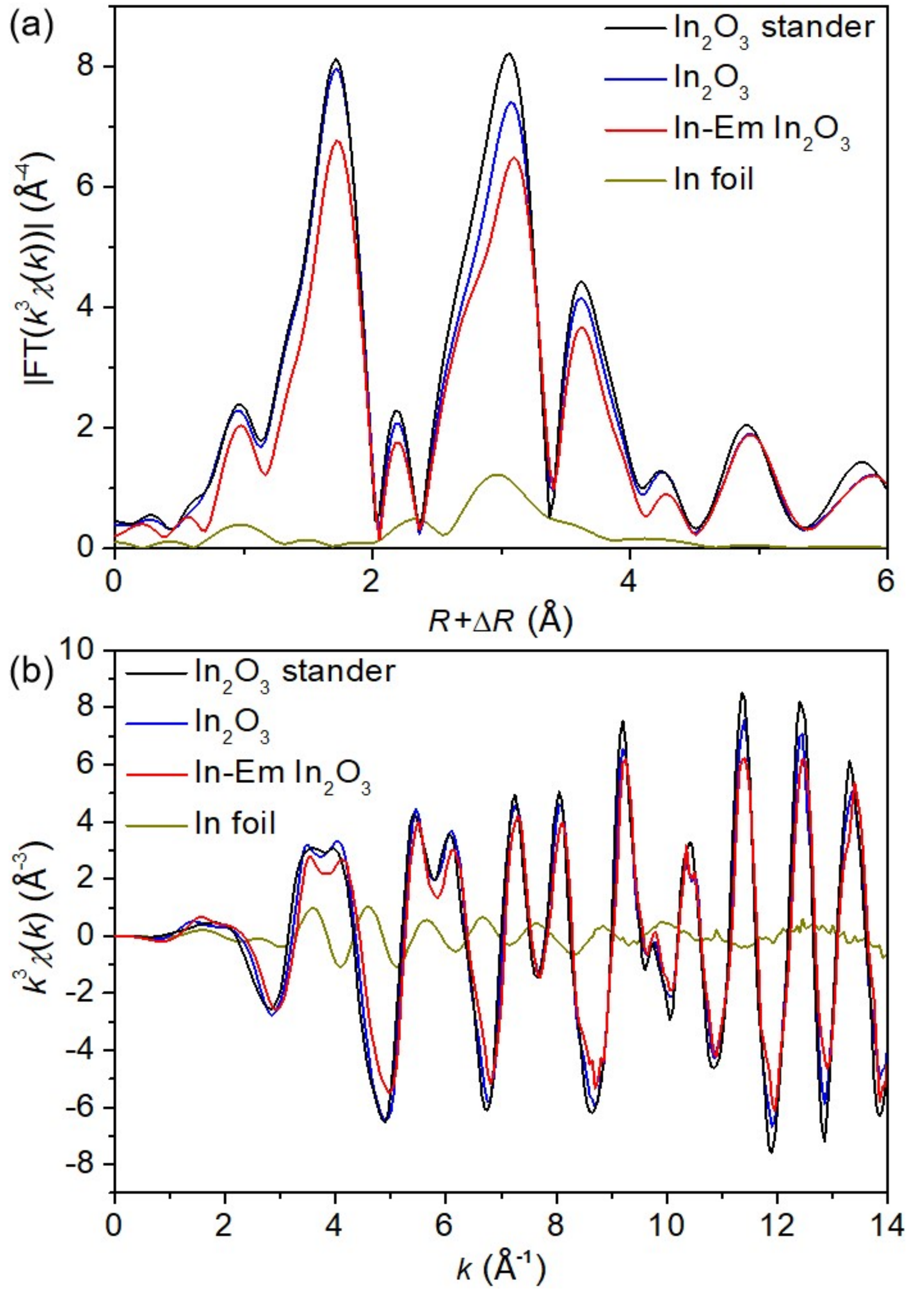

Supplementary Fig. 31 Fourier-transformed **a** R space and **b** K space EXAFS spectra of In. The EXAFS results suggest that there is no significant change in the In-O bond length of the samples and In_2O_3 stander and the In-O and In-In coordination number of the samples is less than standard In_2O_3 stander.

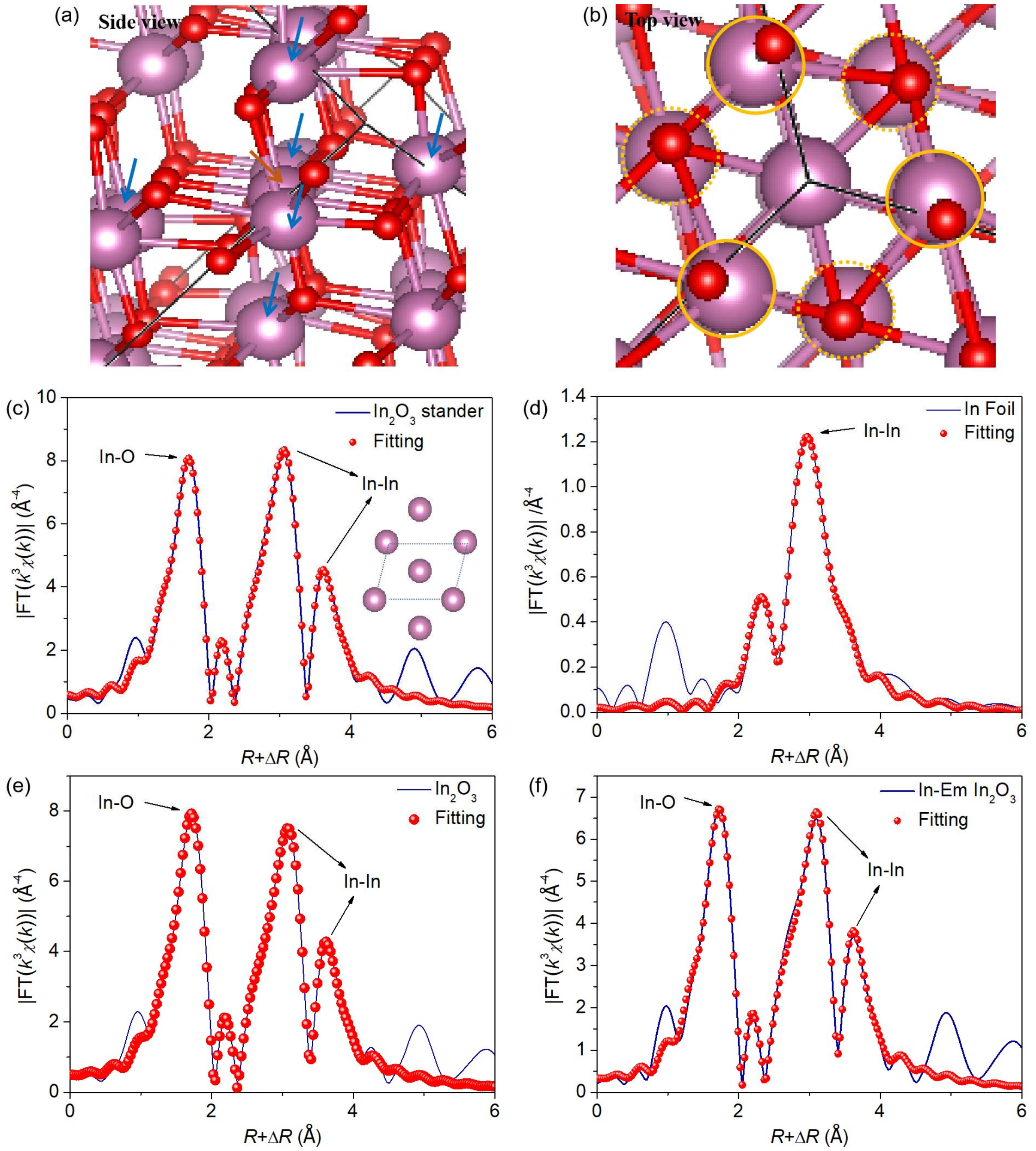

Supplementary Fig. 32 **a** Side view and **b** top view of six-coordination In-In unit. The corresponding R space EXAFS fitting curves of In for **c** In_2O_3 stander, **d** In foil, **e** In_2O_3 and **f** In-Em In_2O_3. Because of the transformation from six- to four-coordination, In density in In-Em In_2O_3 is decreased.

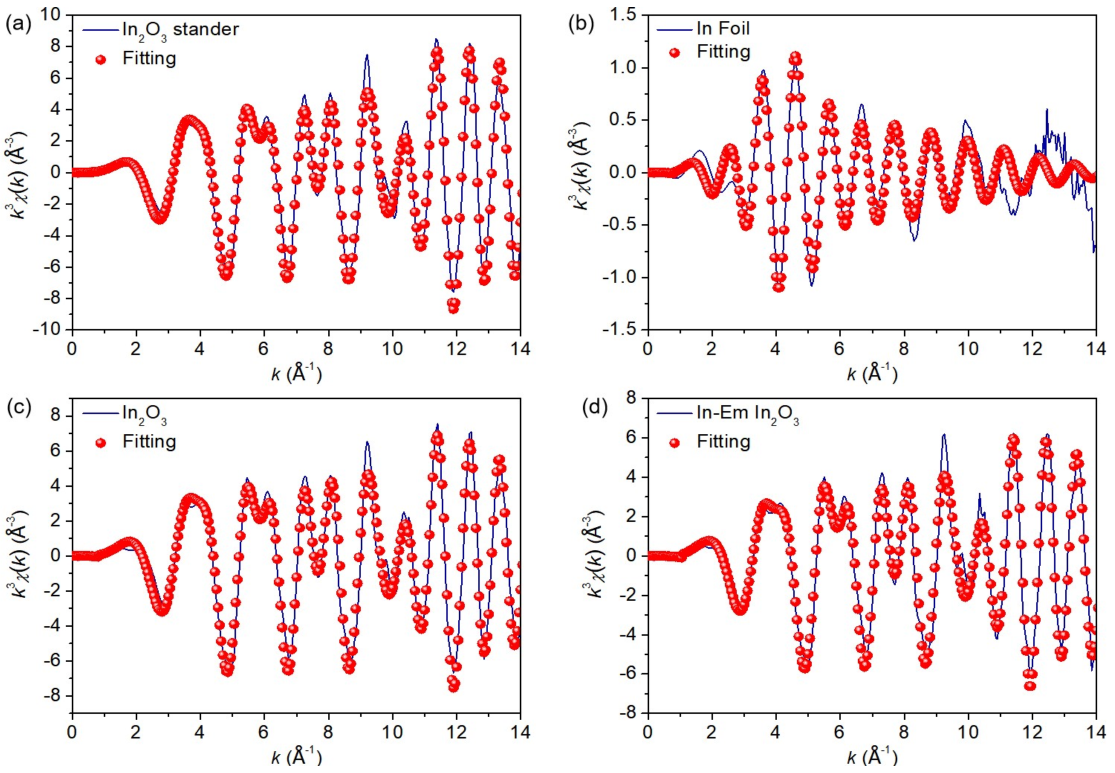

Supplementary Fig. 33 The corresponding K space EXAFS fitting curves of In for **a** In_2O_3 stander, **b** In foil, **c** In_2O_3 and **d** In-Em In_2O_3.

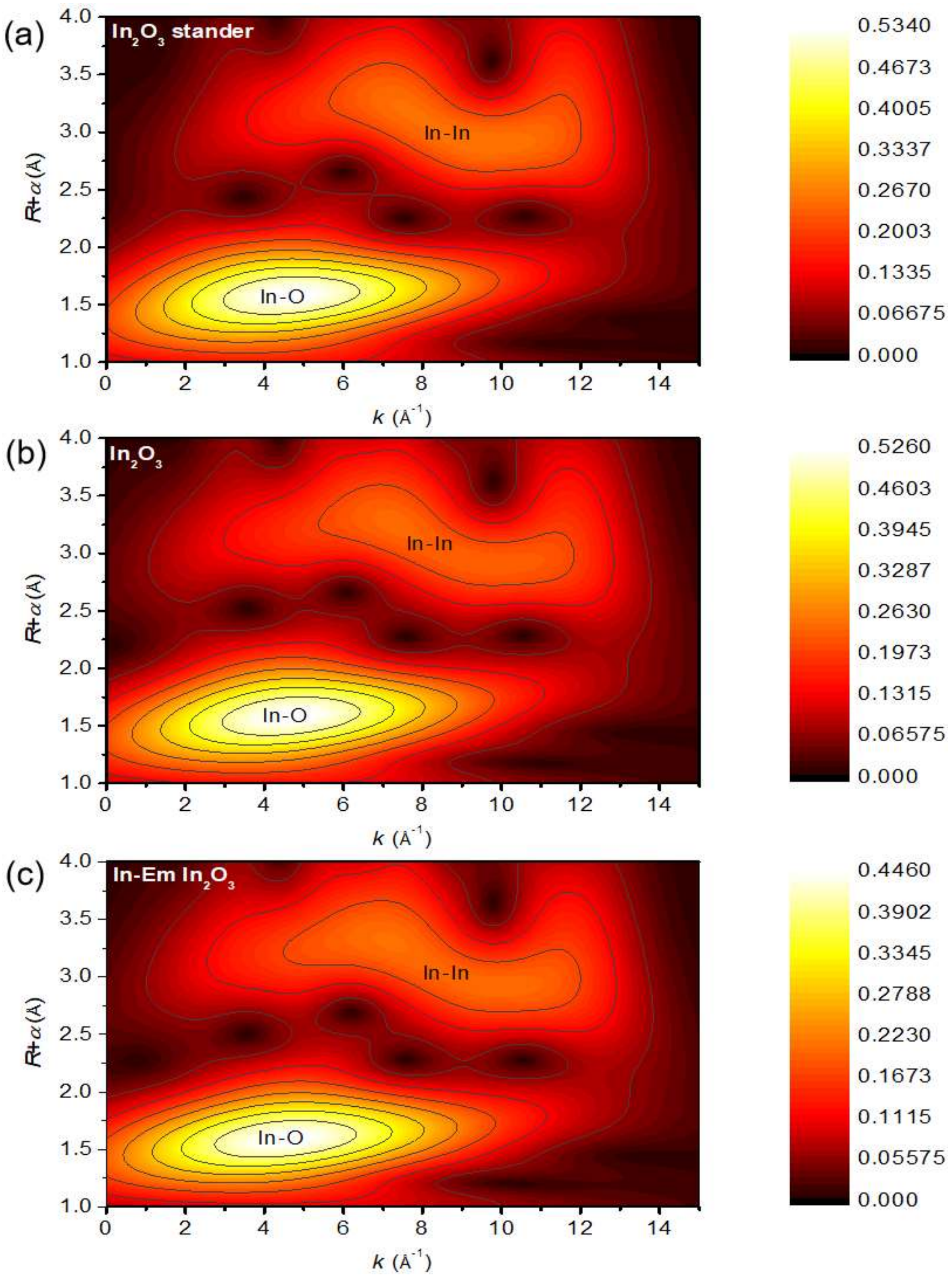

Supplementary Fig. 34 Wavelet transform analysis of **a** In_2O_3 stander, **b** In_2O_3 and **c** In-Em In_2O_3. For Wavelet Transform analysis, the $\chi(k)$ exported from Athena was imported into the Hama Fortran code. The parameters were listed as follow: R range, 1~4.0 Å, k range, 0~13.0 Å^{-1} for the Samples; k weight, 2; and Morlet function with κ= 10, σ= 1 was used as the mother wavelet to provide the overall distribution. The conclusion was consistent with that from the corresponding R space and K space EXAFS. Intensity decreases in the order of red and yellow.

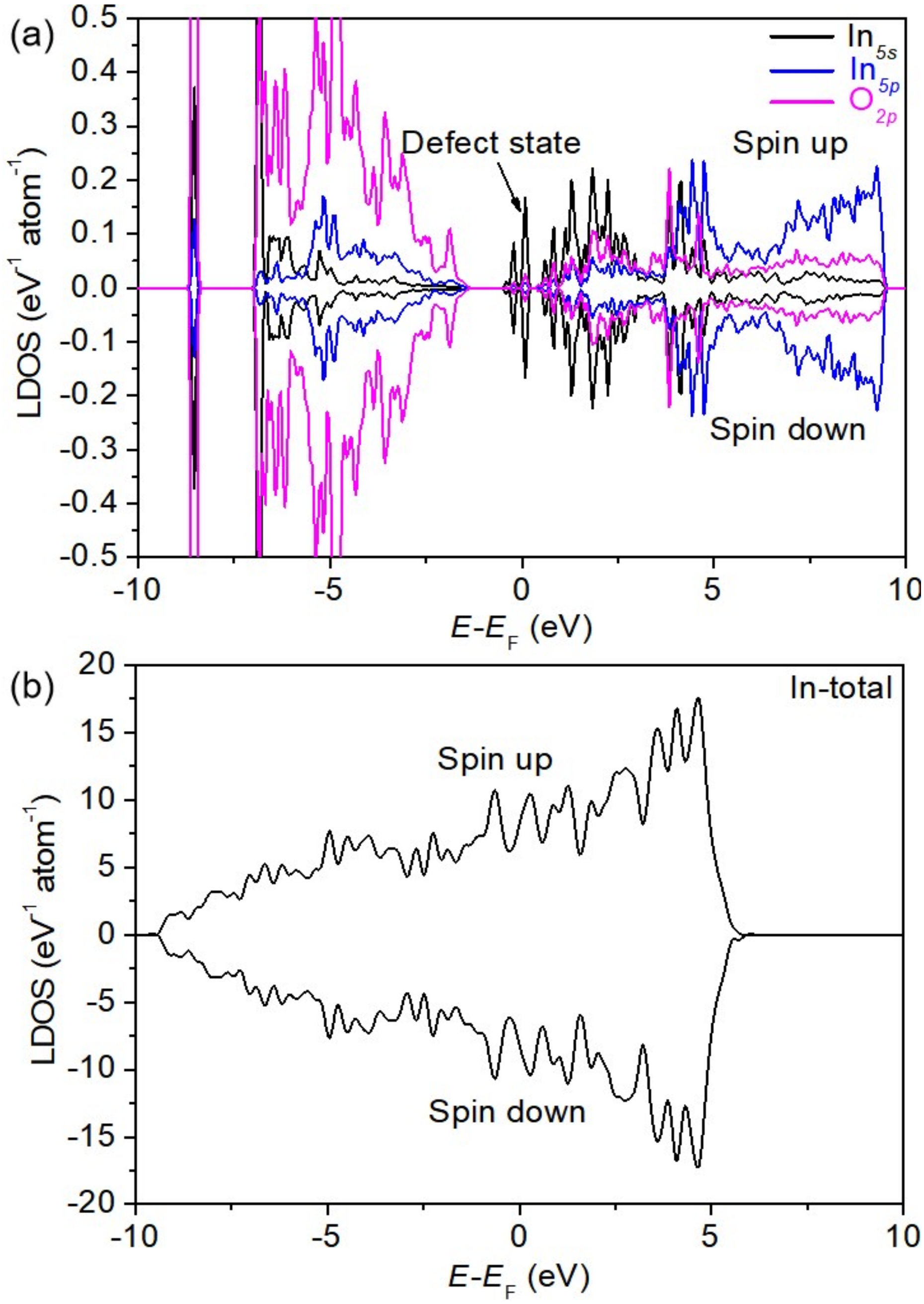

Supplementary Fig. 35 LDOS of **a** defective In_2O_3 and **b** metallic In. For defective In_2O_3, a new defect state emerges in the band gap, which is predominantly contributed by an unoccupied In_{5s} level, corresponding to the O-In-(O)V_o-In-In complex, consistent with the analysis of ESR spectra and positron annihilation spectra. The itinerant electronic behavior across Fermi level reflects the characteristics of electron free-movement in metallic In due to the coupling interaction between In_{3d} electrons.

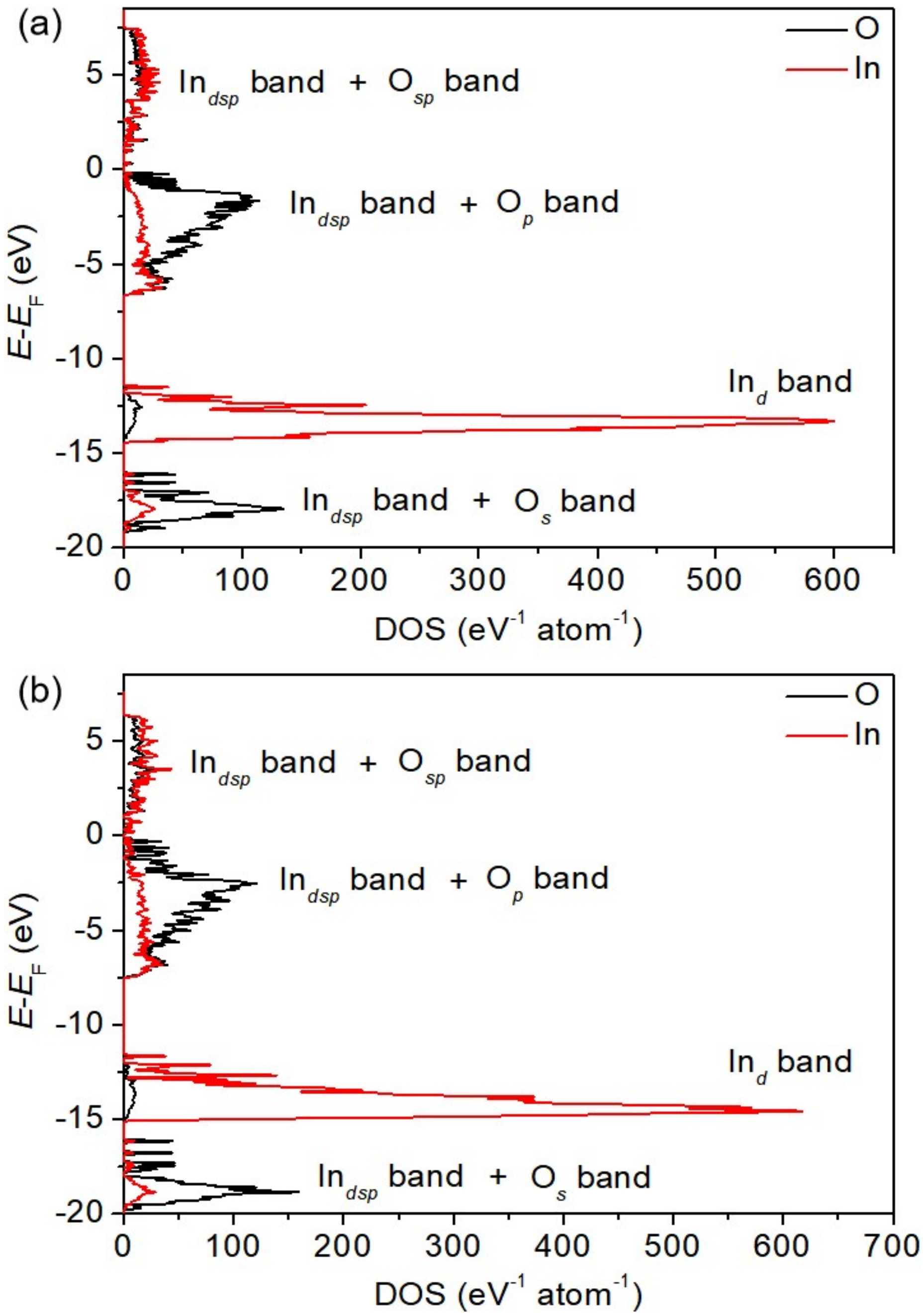

Supplementary Fig. 36 LDOS of **a** In_2O_3 and **b** In-In_2O_3.

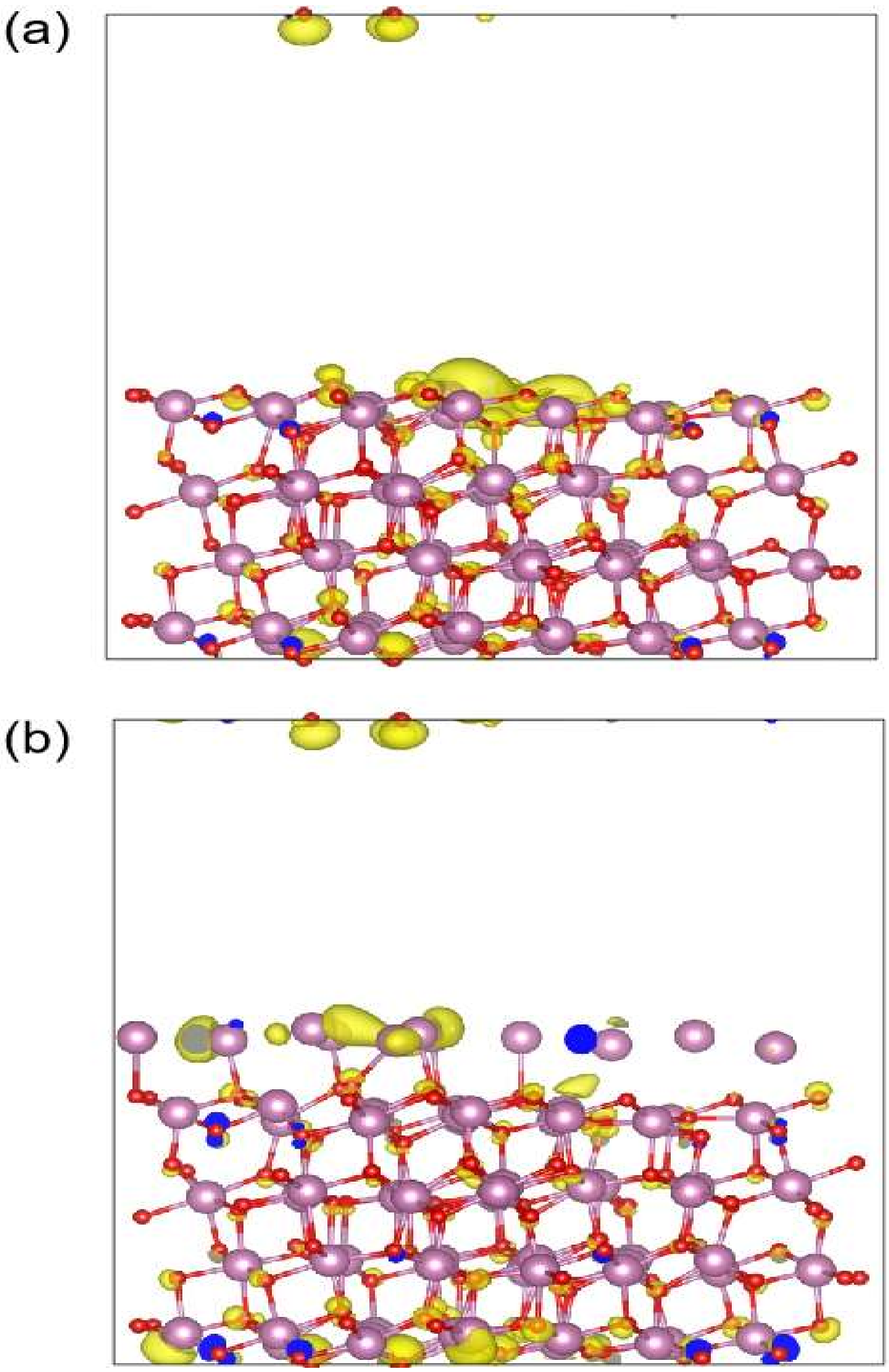

Supplementary Fig. 37 Charge distribution derived from wave function of energy levels at VBM of **a** In_2O_3 and **b** In-In_2O_3.

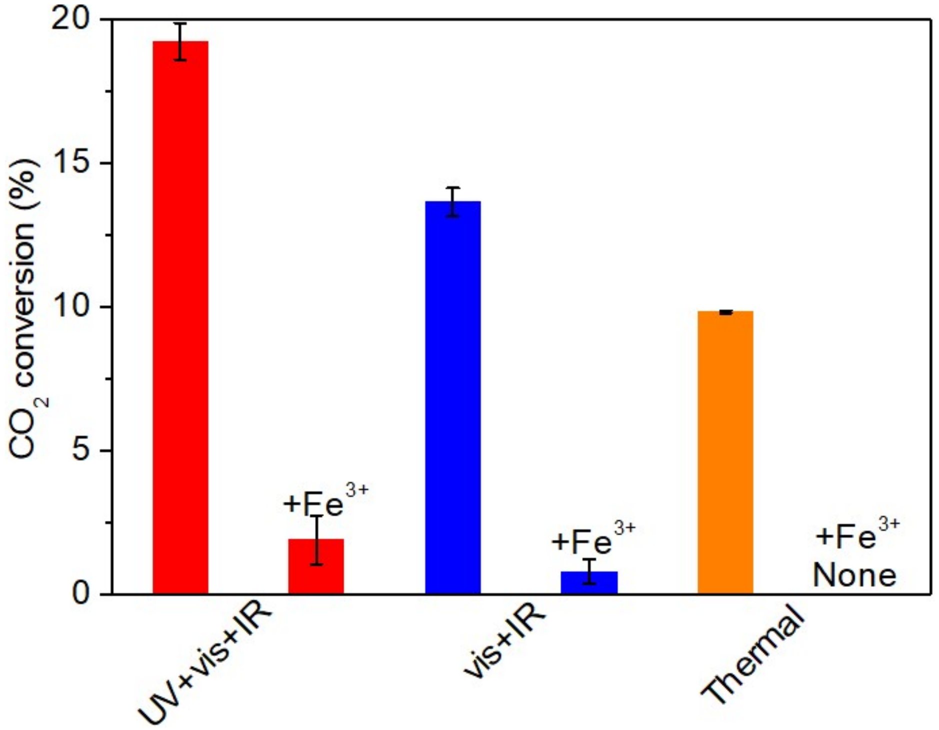

Supplementary Fig. 38 Performances over In-Em In_2O_3 with Fe^{3+} under full-spectrum or vis+IR light irradiation and under solely thermal catalysis. (The error bars represent standard deviation.)

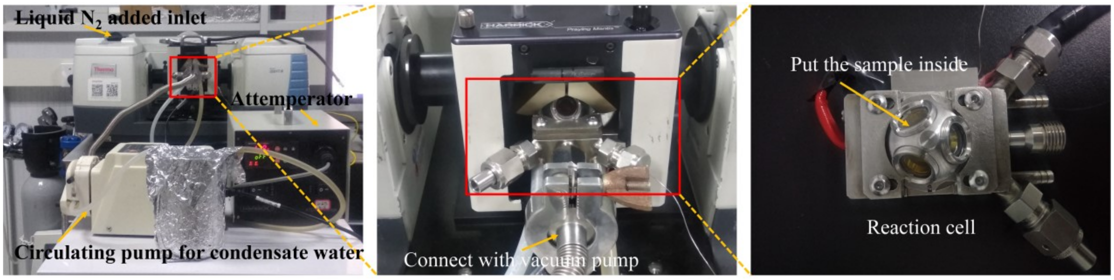

Supplementary Fig. 39 The pictures of Nicolet iS50FTIR spectrometer with a liquid-nitrogen-cooled MCT-A detector and reaction cell.

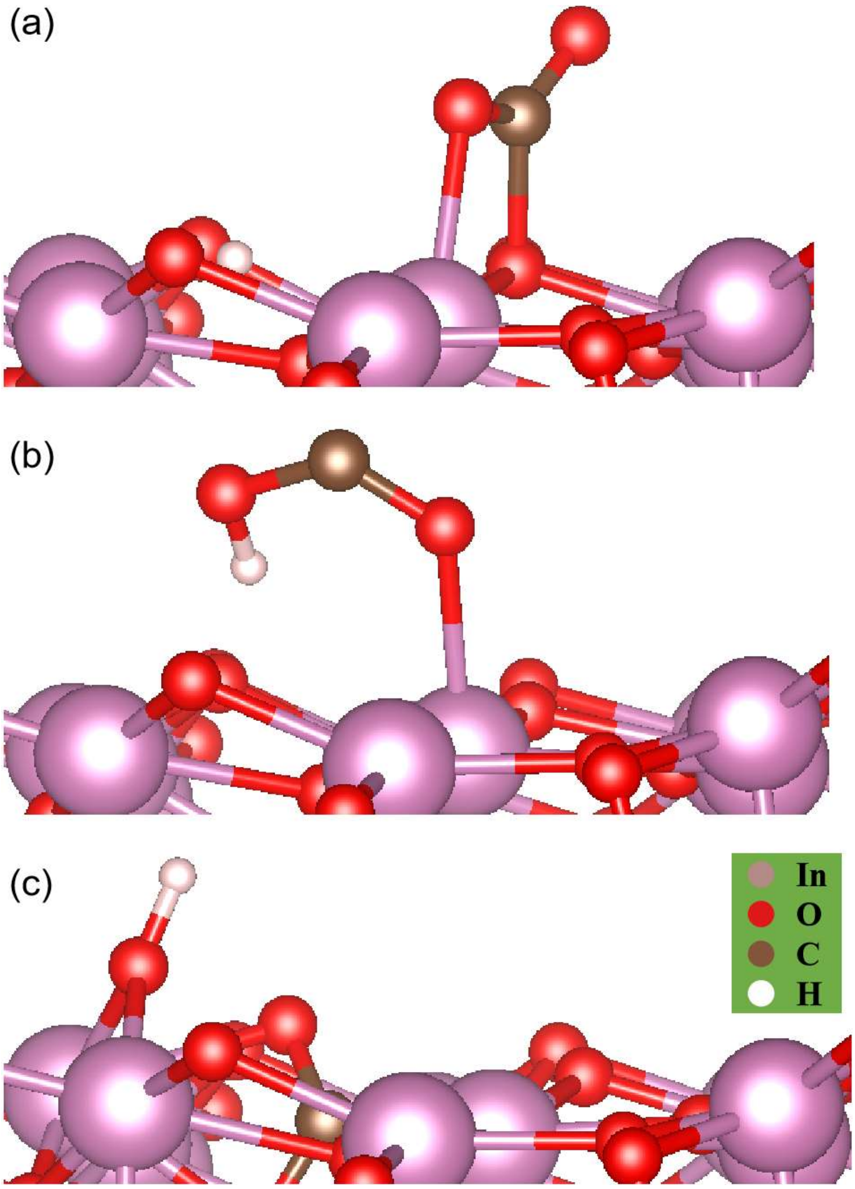

Supplementary Fig. 40 The adsorbate binding configurations on the surface of In_2O_3. **a** Adsorbed CO_2 and H. **b** COOH intermediate. **c** CO and OH from COOH dissociation.

Supplementary Discussion: The C atom and one of the O atoms of CO_2 are bound with O and In of In_2O_3 around oxygen vacancy, respectively. The two C-O bonds of CO_2 form a bond angle of 120°, suggesting CO_2 activation. The bent structure of CO_2 is the feature of CO_2 anion[16]. With the collision coupling of H and the other O atom of CO_2, COOH forms accompanied by the O end linking with In atom. After the dissociation of COOH, the OH is bridged with two In atoms.

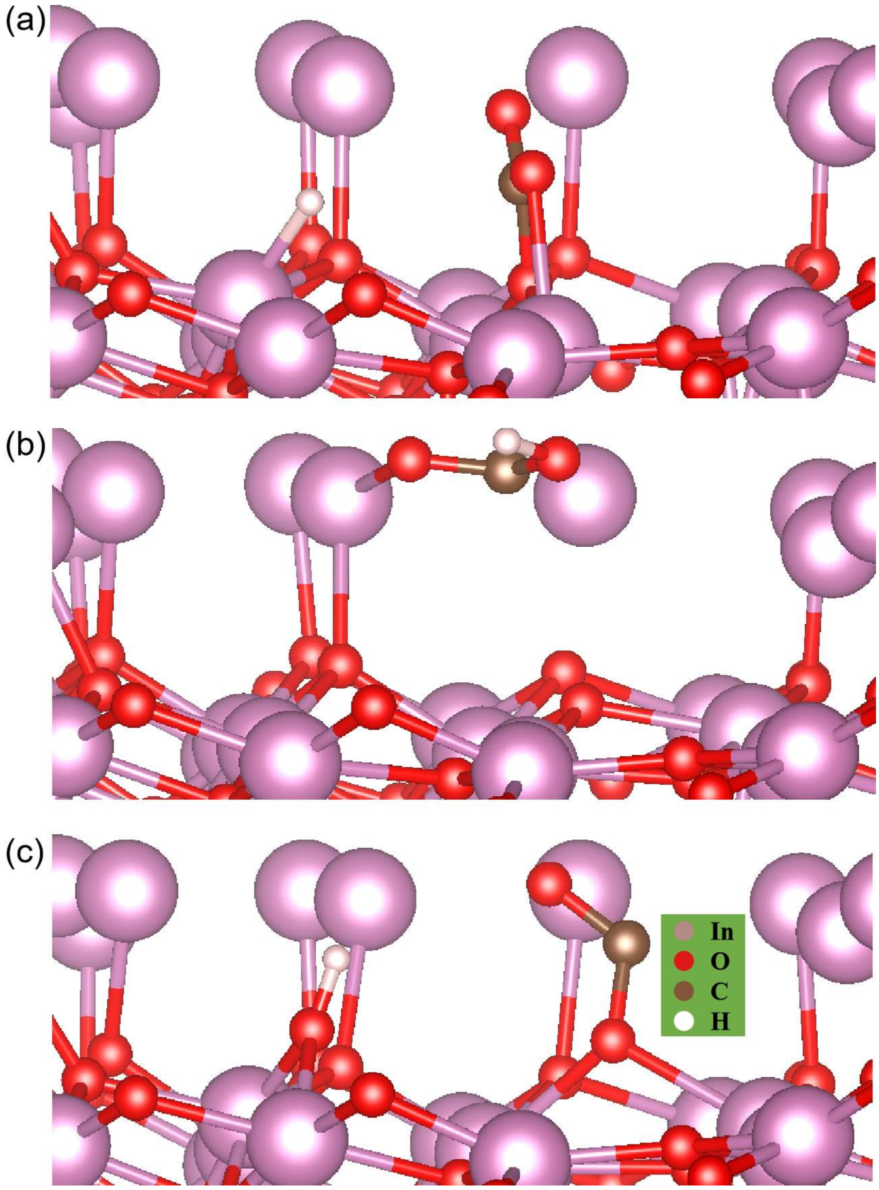

Supplementary Fig. 41 The adsorbate binding configurations on the surface of In-In_2O_3. **a** Adsorbed CO_2 and H. **b** COOH intermediate. **c** CO and OH from COOH dissociation.

Supplementary Discussion: The adsorption of H and CO_2 is on the In_2O_3 side and the interaction mode is similar with the result above of In_2O_3. H and CO_2 collide at the interface to form COOH. One of the O atoms of COOH is connected with In^0 atom. Finally, COOH is dissociated with the OH bridged between two In atoms and the CO linking with O atom.

Supplementary Tables

Supplementary Table 1 Specific surface areas of In_2O_3 and In-Em In_2O_3. By comparing their specific surface areas, the specific surface area of In_2O_3 is much larger than that of In-Em In_2O_3. Hence, it is clear that the specific surface area is not the reason for the enhanced activity.

Catalyst	BET surface area (m^2 g^{-1})	Langmuir surface area (m^2 g^{-1})
In_2O_3	101.25	6.94
In-Em In_2O_3	140.47	9.59

Supplementary Table 2 Through Scherrer formula

$$D=K\gamma/B\cos\theta \quad (17)$$

where D is grain size, K is Scherrer constant-0.89, B is full width at half maxima (*FWHM*), θ is diffraction angle and γ is X-ray wavelength of 0.154056 nm. XRD patterns were used to calculate the D value of every sample. The diffraction peaks of the In_2O_3 crystal facet (222) were used as the calculated diffraction peaks.

Catalyst	*FWHM*	θ (°)	D (nm)
In_2O_3	0.0064	15.32	22.2
In-Em In_2O_3	0.0061	15.31	23.2

Supplementary Table 3 Lifetimes (τ) and relative intensities (I) of positrons in positron annihilation spectra of In_2O_3 and In-Em In_2O_3, obtained from three-component fitting analysis.

Catalyst	τ_1 (ns)	I_1 (%)	τ_2 (ns)	I_2 (%)	τ_3 (ns)	I_3 (%)	$\tau_{average}$ (ns)	Goodness of Fit
In_2O_3	0.229	22.7	0.406	75.9	2.766	1.4	0.399	1.00
In-Em In_2O_3	0.220	76.7	0.527	20.0	6.173	3.3	0.478	1.15

According to the literature[17], the average lifetime ($\tau_{average}$) can be calculated through

$$\tau_{average}= \tau_1 I_1 * \tau_2 I_2 * \tau_3 I_3 \quad (18)$$

Supplementary Table 4 Performance comparison of different catalysts for CO_2 hydrogenation into CO.

Catalyst	Reaction temp. (°C)	CO_2/ H_2	TOF (h^{-1})	Catalytic type	Ref.
In-Em In_2O_3	300, 380	1/3	2990, 7615	Photothermal catalysis	This work
In_2O_3 NPs	330	1/3	17	Photothermal catalysis	-
In-In_2O_3 NPs	360	1/3	197	Photothermal catalysis	-
Black In_2O_{3-x}	370	1/1	1084[a]	Photothermal catalysis	[18]
Black In_2O_{3-x}	300	1/1	1152[b]	Photothermal catalysis	[19]
$In_2O_{3-x}(OH)_y$	150	1/1	0.001[a]	Photothermal catalysis	[20]
c-In_2O_3	350	1/2	1764[c]	Thermal catalysis	[21]
h-In_2O_3	350	1/2	1638[c]	Thermal catalysis	[21]
$In_2O_{3-x}(OH)_y$ superstructure	R. T.	1/1	0.17[a]	Photocatalysis	[22]
Bi_2O_{3-x}	200	1/2	280[a]	Photothermal catalysis	[23]
(X)Ni-In_2O_3 (X: 1, 5, 10, 15)	280	1/4	10, 14, 17, 41[a]	Thermal catalysis	[24]
In_2O_3/ZrO_2	250	1/3	0.84[a]	Thermal catalysis	[25]
In_2O_3/CeO_2-h	250	1/3	22[a]	Thermal catalysis	[25]
2Y8In/ZrO_2	300	1/4	0.71[a]	Thermal catalysis	[26]
3La10In/ZrO_2	300	1/4	0.92[a]	Thermal catalysis	[26]
Ru/TiO_2	200	3/1	14[d]	Thermal catalysis	[27]
Ni/SiO_2	350	1/4	32[d]	Thermal catalysis	[28]
Pd/ZnO	250	1/3	1[d]	Thermal catalysis	[29]
Ni/HY (molecular sieve)	300	1/4	157[d]	Thermal catalysis	[30]
PtCo/TiO_2	300	1/2	2093[d]	Thermal catalysis	[31]
[PPN] [$RuCl_3(CO)_3$]	160	1/3	17~19	Thermal catalysis	[32]
$CoZrO_x$	340	1/4	265[a]	Thermal catalysis	[33]
Ru/SiO_2	300	1/1	342[d]	Thermal catalysis	[34]

Pt_1/TiO_2	250	1/4	1296[d]	Thermal catalysis	[35]
Pd/CeO_2	275	1/3	200~500[d]	Thermal catalysis	[36]
Rh/Fe_3O_4	300	1/4	468[d], 312[a]	Thermal catalysis	[37]
ZrO_2@Pd/SiO_2	450	1/3	3137[d]	Thermal catalysis	[38]
Ru/CeO_2	330	1/4	1008[d]	Thermal catalysis	[39]

[a] The value in the literature is equal to CO production rate/number of active sites where number of active sites was obtained from CO_2-TPD tests. [b] The value originates from the literature wherein number of active sites was estimated from XPS spectra. [c] The value is estimated afterward wherein the number of active sites was obtained according to the CO_2-TPD tests in the literature. [d] The active sites are reported to be metal catalyst dispersed on oxide and the number is measured via FT-IR and H_2-TPR.

Supplementary Table 5 CO_2 amount adsorbed measured from CO_2-TPD patterns.

Catalyst	In-Em In_2O_3	In-Em In_2O_3-spent(1)	In-Em In_2O_3-spent(10)
Amount adsorbed of CO_2 (mmol g^{-1})	~0.0015	~0.0018	0.062

Supplementary Table 6 EXAFS fitting parameters at the In K-edge for various samples (S_0^2=0.900).

<table>
<tr><th>Sample</th><th>Shell</th><th>C.N.^a</th><th>R (Å)^b</th><th>σ^2 (Å^2)^c</th><th>ΔE_0 (eV)^d</th><th>R factor</th></tr>
<tr><td colspan="7">In K-edge</td></tr>
<tr><td rowspan="2">In foil</td><td>In-In</td><td>4*</td><td>3.14±0.01</td><td>0.0134±0.0025</td><td rowspan="2">2.7±0.8</td><td rowspan="2">0.0024</td></tr>
<tr><td>In-In</td><td>8*</td><td>3.29±0.01</td><td>0.0222±0.0056</td></tr>
<tr><td rowspan="3">In_2O_3 Stander</td><td>In-O</td><td>6.0</td><td>2.16±0.01</td><td>0.0066±0.0006</td><td rowspan="3">0.9±0.6</td><td rowspan="3">0.0029</td></tr>
<tr><td>In-In</td><td>6.0</td><td>3.34±0.01</td><td rowspan="2">0.0032±0.0026</td></tr>
<tr><td>In-In</td><td>3.0</td><td>3.84±0.01</td></tr>
<tr><td rowspan="3">In_2O_3</td><td>In-O</td><td>5.7±0.3</td><td>2.16±0.01</td><td>0.0066±0.0005</td><td rowspan="3">2.5±0.5</td><td rowspan="3">0.0021</td></tr>
<tr><td>In-In</td><td>5.0±0.9</td><td>3.35±0.02</td><td rowspan="2">0.0031±0.0025</td></tr>
<tr><td>In-In</td><td>2.67±1.0</td><td>3.84±0.01</td></tr>
<tr><td rowspan="3">In-Em In_2O_3</td><td>In-O</td><td>4.9±0.2</td><td>2.16±0.01</td><td>0.0063±0.0007</td><td rowspan="3">4.2±0.5</td><td rowspan="3">0.0045</td></tr>
<tr><td>In-In</td><td>3.9±0.7</td><td>3.36±0.01</td><td>0.0059±0.0005</td></tr>
<tr><td>In-In</td><td>2.4±0.6</td><td>3.84±0.01</td><td>0.0030±0.0015</td></tr>
</table>

[a]*C.N.*, coordination number; [b]*R*, distance between absorber and back scatter atoms; [c]σ^2, Debye-Waller factor to account for both thermal and structural disorders; [d]ΔE_0, inner potential correction; *R* factor indicates the goodness of the fit. S_0^2 was fixed to 0.900, according to the experimental EXAFS fit of In foil by fixing *C.N.* as the known crystallographic value. Fitting range: $3.0 \leq k$ (/Å) ≤ 10.5 and $1.7 \leq R$ (Å) ≤ 3.5 (In foil); $3.0 \leq k$ (/Å) ≤ 12.5 and $1.0 \leq R$ (Å) ≤ 4.0 (In_2O_3 Stander, In_2O_3 and In-Em In_2O_3). A reasonable range of EXAFS fitting parameters: $0.700 < S_0^2 < 1.000$; *C.N.* > 0; $\sigma^2 > 0$ Å^2; $\Delta E_0 < 10$ eV; *R* factor < 0.02.

Supplementary References

1. Pulido, A. et al. Combined DFT/CC and IR spectroscopic studies on carbon dioxide adsorption on the zeolite H-FER. *Energy Environ. Sci.* **2**, 1187−1195 (2009).
2. Fujimori, K. et al. Visualizing cation vacancies in Ce:$Gd_3Al_2Ga_3O_{12}$ scintillators by gamma-ray-induced positron annihilation lifetime spectroscopy. *Appl. Phys. Express* **13**, 085505 (2020).
3. Kresse, G. Efficient iterative schemes for ab initio total-energy calculations using a plane-wave basis set. *Phys. Rev. B* **54**, 11169−11186 (1996).
4. Kresse, G. & Furthmueller, J. Efficiency of ab-initio total energy calculations for metals and semiconductors using a plane-wave basis set. *Comput. Mater. Sci.* **6**, 15−50 (1996).
5. Perdew, J. P., Burke, K. & Ernzerhof, M. Generalized gradient approximation made simple. *Phys. Rev. Lett.* **77**, 3865−3868 (1996).
6. Blöchl, P. E. Projector augmented-wave method. *Phys. Rev. B* **50**, 17953−17979 (1994).
7. Gao, P. et al. Direct conversion of CO_2 into liquid fuels with high selectivity over a bifunctional catalyst. *Nat. Chem.* **9**, 1019−1024 (2017).
8. Qin, B. & Li, S. First principles investigation of dissociative adsorption of H_2 during CO_2 hydrogenation over cubic and hexagonal In_2O_3 catalysts. *Phys. Chem. Chem. Phys.* **22**, 3390−3399 (2020).
9. Ye, J., Liu, C. & Ge, Q. DFT study of CO_2 adsorption and hydrogenation on the In_2O_3 surface. *J. Phys. Chem. C* **116**, 7817−7825 (2012).
10. Monkhorst, H. J. & Pack, J. D. Special points for brillouin-zone integrations. *Phys. Rev. B* **13**, 5188−5192 (1976).
11. Dutta, D., Sudarsan, V., Srinivasu, P., Vinu, A. & Tyagi, A. Indium oxide and europium/dysprosium doped indium oxide nanoparticles: sonochemical synthesis, characterization, and photoluminescence studies. *J. Phys. Chem. C* **112**, 6781−6785 (2008).
12. Parent, P., Dexpert, H. & Tourillon, G. Structural study of tin-doped indium oxide thin films using X-ray absorption spectroscopy and X-ray diffraction. *J. Electrochem. Soc.* **139**, 276−281 (1992).
13. Baszczuk, A. et al. Luminescence properties of europium activated $SrIn_2O_4$. *J. Alloys Compd.* **394**, 88−92 (2005).
14. Kaur, M. et al. Room-temperature H_2S gas sensing at ppb level by single crystal In_2O_3 whiskers. *Sens. Actuators, B* **133**, 456−461 (2008).
15. Gurlo, A. et al. Pressure-induced decomposition of indium hydroxide. *J. Am. Chem. Soc.* **132**, 12674−12678 (2010).
16. Sakaki, S. & Dedieu, A. Coordination mode and bonding nature of carbon dioxide in d8 $[Co(alcn)_2(CO_2)]^-$ (alcn= $HNCHCHCHO^-$). An ab initio MO study. *Inorg. Chem.* **26**, 3278-3284 (1987).
17. Dutta, S. et al. Annealing effect on nano-ZnO powder studied from positron lifetime and optical absorption spectroscopy. *J. Appl. Phys.* **100**, 114328 (2006).
18. Qi, Y. et al. Photoinduced defect engineering: enhanced photothermal catalytic performance of 2D black In_2O_{3-x} nanosheets with bifunctional oxygen vacancies. *Adv. Mater.* **32**, 1903915 (2020).
19. Wang, L. et al. Black indium oxide a photothermal CO_2 hydrogenation catalyst. *Nat.*

Commun. **11**, 2432 (2020).

20. Hoch, L. B. et al. The rational design of a single-component photocatalyst for gas-phase CO_2 reduction using both UV and visible light. *Adv. Sci.* **1**, 1400013 (2014).
21. Wang, J. et al. Variation in the In_2O_3 crystal phase alters catalytic performance toward the reverse water gas shift reaction. *ACS Catal.* **10**, 3264−3273 (2019).
22. He, L. et al. Spatial separation of charge carriers in $In_2O_{3-x}(OH)_y$ nanocrystal superstructures for enhanced gas-phase photocatalytic activity. *ACS Nano* **10**, 5578−5586 (2016).
23. Li, Y. et al. Plasmonic hot electrons from oxygen vacancies for infrared light-driven catalytic CO_2 reduction on Bi_2O_{3-x}. *Angew. Chem. Int. Ed.* **60**, 910−916 (2021).
24. Frei, M. S. et al. Nanostructure of nickel-promoted indium oxide catalysts drives selectivity in CO_2 hydrogenation. Nat. *Commun.* **12**, 1960 (2021).
25. Regalado Vera, C. Y. et al. Mechanistic understanding of support effect on the activity and selectivity of indium oxide catalysts for CO_2 hydrogenation. *Chem. Eng. J.* **426**, 131764 (2021).
26. Chou, C. Y. & Lobo, R. F. Direct conversion of CO_2 into methanol over promoted indium oxide-based catalysts. *Appl. Catal., A* **583**, 117144 (2019).
27. Matsubu, J. C., Yang, V. N. & Christopher, P. Isolated metal active site concentration and stability control catalytic CO_2 reduction selectivity. *J. Am. Chem. Soc.* **137**, 3076−3084 (2015).
28. Aldana, P. A. U. et al. Catalytic CO_2 valorization into CH_4 on Ni-based ceria-zirconia. reaction mechanism by operando IR spectroscopy. *Catal. Today* **215**, 201−207 (2013).
29. Bahruji, H. et al. Pd/ZnO catalysts for direct CO_2 hydrogenation to methanol. *J. Catal.* **343**, 133−146 (2016).
30. Aziz, M. A. A. et al. Highly active Ni-promoted mesostructured silica nanoparticles for CO_2 methanation. *Appl. Catal., B* **147**, 359−368 (2014).
31. Kattel, S. et al. CO_2 Hydrogenation over oxide-supported PtCo catalysts: the role of the oxide support in determining the product selectivity. *Angew. Chem. Int. Ed.* **55**, 7968−7973 (2016).
32. Tsuchiya, K., Huang, J. D. & Tominaga, K. I. Reverse water-gas shift reaction catalyzed by mononuclear Ru complexes. *ACS Catal.* **3**, 2865−2868 (2013).
33. Dostagir, N. H. M. et al. Co single atoms in ZrO_2 with inherent oxygen vacancies for selective hydrogenation of CO_2 to CO. *ACS Catal.* **11**, 9450−9461 (2021).
34. Mansour, H. & Iglesia, E. Mechanistic connections between CO_2 and CO hydrogenation on dispersed ruthenium nanoparticles. *J. Am. Chem. Soc.* **143**, 11582−11594 (2021).
35. Chen, L. et al. Unlocking the catalytic potential of TiO_2-supported Pt single atoms for the reverse water-gas shift reaction by altering their chemical environment. *J. Am. Chem. Soc. (Au)* **1**, 977−986 (2021).
36. Cao, F. et al. Size-controlled synthesis of Pd nanocatalysts on defect-engineered CeO_2 for CO_2 hydrogenation. *ACS Appl. Mater. Inter.* **13**, 24957−24965 (2021).
37. Zhu, Y. et al. Environment of metal-O-Fe bonds enabling high activity in CO_2 reduction on single metal atoms and on supported nanoparticles. *J. Am. Chem. Soc.* **143**, 5540−5549 (2021).
38. Du, Y. P. et al. Engineering the ZrO_2-Pd interface for selective CO_2 hydrogenation by overcoating an atomically dispersed Pd precatalyst. *ACS Catal.* **10**, 12058−12070 (2020).
39. Wang, Y. et al. Site-selective CO_2 reduction over highly dispersed Ru-SnO_x sites derived

from a [Ru@Sn_9]$_6$-zintl cluster. *ACS Catal.* **10**, 7808−7819 (2020).
